# OsKAT1 is a short Shaker potassium channel involved in root-to-shoot potassium translocation and contributes to rice grain yield

**DOI:** 10.1073/pnas.2527650123

**Published:** 2026-01-28

**Authors:** Shunying Yang, Thanh-Hao Nguyen, Cécile Fizames, Junlin Li, Sheliang Wang, Aurore Vernet, Emmanuel Guiderdoni, Shaofei Wang, Yixiu Guo, Weiwei Zhang, Tianqi Wei, Yanan Huang, Dongli Hao, Jiajin Wang, Hervé Sentenac, Anne-Aliénor Véry, Ren Fang Shen, Yanhua Su

**Affiliations:** ^a^State Key Laboratory of Soil and Sustainable Agriculture, Institute of Soil Science, Chinese Academy of Sciences, Nanjing 211135, China; ^b^Institute for Plant Sciences of Montpellier, University of Montpellier, Centre National de la Recherche Scientifique (Unité Mixte de Recherche 5004), Institut national de recherche pour l’agriculture, l’alimentation et l’environnement (Unité Mixte de Recherche 386), Institut Agro, Montpellier 34060, France; ^c^Shandong Institute of Sericulture, Yantai 264002, China; ^d^National Key Laboratory of Crop Genetic Improvement, Huazhong Agricultural University, Wuhan 430070, China; ^e^FranceCentre de coopération internationale en recherche agronomique pour le développement (CIRAD), Unité Mixte de Recherche Amélioration Génétique et Adaptation des Plantes Méditerranéennes et Tropicales Institut, Montpellier F-34398, France; ^f^University of Montpellier, Centre de coopération internationale en recherche agronomique pour le développement (CIRAD), Institut national de recherche pour l’agriculture, l’alimentation et l’environnement (INRAE), Institut Agro, Montpellier F-34398, France; ^g^University of Chinese Academy of Sciences, Beijing 100049, China; ^h^College of Ecology and Environment, Nanjing Forestry University, Nanjing 210037, China; ^i^Jiangsu Key Laboratory for the Research and Utilization of Plant Resources, Institute of Botany, Jiangsu Province and Chinese Academy of Sciences (Nanjing Botanical Garden Mem. Sun Yat-Sen), Nanjing 210014, China; ^j^University of Chinese Academy of Sciences, Nanjing 211135, China

**Keywords:** short Shaker channel, root stele, root-to-shoot K^+^ transport, rice grain yield

## Abstract

Shaker potassium channels play essential roles in K^+^ uptake and distribution in plants. Studies on Shaker channels in *Arabidopsis* have provided a paradigmatic framework, but the rice genome encodes an additional member, OsKAT1, whose function remians poorly defined. OsKAT1-type channels are characterized by an innately short cytosolic C terminus, forming a unique clade of monocot-specific short Shakers that is prevalent in *Poaceae* species. In rice, OsKAT1 is predominantly expressed in the root stele. Disruption of OsKAT1 (KO mutation) leads to a significant reduction in K^+^ secretion into the xylem sap delivered to the shoot. Patch-clamp experiments on root stele protoplasts of WT and KO plants indicate that OsKAT1 functions as an inward channel. Functional analyses in *Xenopus* oocytes reveal that despite activating at weakly negative voltages, OsKAT1 is intrinsically incapable of mediating substantial outward currents—a property attributed to its truncated C terminus. Together with outward K^+^ channel activity, this feature enables stelar cells to remain significantly permeable to K^+^ around the K^+^ equilibrium potential. When expressed in *Arabidopsis*, OsKAT1 contributes to K^+^ transport in the xylem sap, but only in wild-type plants that express SKOR, a Shaker channel specialized for this role. These results demonstrate that OsKAT1 constitutes a component of K^+^ translocation to shoots and shed light on the energization and regulation underlying this function. Moreover, OsKAT1 is shown to improve rice adaptation to environmental conditions and enhance grain yield under field conditions through its facilitating root-to-shoot ion translocation.

## Significance

K^+^ transport from root to shoot is a critical process for sustaining physiological functions in aerial organs. We report here a short inward channel OsKAT1, localized in the root stele of rice, which contributes to root–shoot K^+^ translocation. Functional comparison between OsKAT1 and its C-terminal extended chimera OsKAT1-C1 in both *Xenopus* oocytes and *Arabidopsis* roots demonstrates that the short channel OsKAT1 mediates more efficient K^+^ transport than the chimera carrying a canonical C terminus. Under field conditions, OsKAT1 activity shows significant contributions to K^+^ accumulation in shoots and grain yield of rice, highlighting its agronomic importance.

K^+^ is the most abundant cation in plants and plays a vital role in multiple aspects of plant growth and development. It is involved in regulating cellular osmotic pressure, controlling of stomatal movement and enhancing plant resistance to both abiotic and biotic stresses (1–3). To support these functions, a substantial amount of K^+^ must be transported via the xylem from roots to aerial parts of the plants (2, 3).

K^+^ uptake at the root surface constitutes the initial step in the long-distance translocation of this ion from root to shoot. This process is mediated by both high-affinity transporters, such as members of the HAK/KUP/KT family (4–9), and Shaker-type channels like AKT1 (10–12). Following uptake, K^+^ moves centripetally across root tissues (e.g., cortex and endodermis) toward the stele. This radial transport occurs *via* symplastic pathways through plasmodesmata and/or apoplastic routes, facilitated by specific transporter proteins that enable cell-to-cell transfer. Notably, some HAK/KUP/KT transporters, which exhibit broad tissue localization, contribute to this centripetal movement and promote K^+^ delivery to the stele (13–16). Subsequently, K^+^ is loaded into the xylem vessels (the central conduits for long-distance transport) primarily via channel-mediated K^+^ efflux from the surrounding stele cells. In the root stele cells, both inward- and outward-rectifying channel currents have been evidenced in barley (17), maize (18, 19), and *Arabidopsis* (20). In *Arabidopsis*, the outward-rectifying Shaker K^+^ channel SKOR is localized to the root stele and is responsible for secreting K^+^ from these stele cells (including the pericycle and xylem parenchyma cells) into the xylem vessels. Loss of SKOR function decreases the K^+^ concentration in the xylem sap and reduces shoot K^+^ content by approximately 50%, highlighting its essential role in the long-distance transport of K^+^ (21). Orthologs of the *SKOR* gene appear to be present in all plant genomes annotated so far (PlaBiPD, https://www.plabipd.de), suggesting that the SKOR-mediated pathway represents a conserved mechanism for K^+^ transport from root to shoot. This functional conservation is further supported by the roles of outward-rectifying K^+^ channels such as OsK5.2 in rice (22, 23) and SlSKOR in tomato (24), both of which have been shown to facilitate long-distance K^+^ translocation. Nevertheless, the molecular identity and physiological role of the inward-rectifying K^+^ channel current in the root stele cells remain unknown.

In plants, the apoplastic K^+^ concentrations are approximately one to two orders of magnitude lower than the cytoplasmic levels (25). This steep concentration gradient contributes to a physiological membrane potential that is typically more negative than −60 mV. Thereby, plasma membrane depolarization is required for activating SKOR-like channels to mediate K^+^ secretion from stelar cells. The molecular mechanisms underlying the energetics of xylem loading that enable long-distance K^+^ translocation have been recently discussed (26). The membrane potential of xylem parenchyma cells is estimated to range from −50 to −150 mV. Under depolarized conditions (e.g., near −50 mV), S‐type anion channels such as SLAH2 and SLAH3 in *Arabidopsis* become active, which both tend to further depolarize the membrane potential and favors anion translocation toward the shoot. In contrast, under strongly hyperpolarized potentials, K^+^ accumulation into parenchyma cells is thought to be facilitated either by inward-rectifying K^+^ channels in a voltage-dependent manner or by KUP/KT/HAK-type H^+^-K^+^ cotransporters (26).

The Shaker channel family is highly conserved in plants, with each genome encoding approximately 10 members that can be classified into 5 subfamilies (5). Homologs of the *Arabidopsis* KAT1/KAT2, GORK, AKT2, and AKT1 have been identified not only in rice (5, 12, 22, 23, 27, 28) but also in numerous other dicot and monocot species, such as potato (29), grapevine (30, 31), maize (32, 33), barley (34), poplar (35), and the desert shrub *Ammopiptanthus mongolicus* (36, 37). In *Arabidopsis*, KAT1 and KAT2 mediate K^+^ influx into guard cells to promote stomatal opening, whereas GORK facilitates K^+^ efflux from these cells, leading to stomatal closure (38–41). AKT2 plays a key role in K^+^ redistribution and recirculation within the phloem vasculature (27, 42, 43). In roots, AKT1 is responsible for K^+^ uptake from the soil solution (10, 11), while SKOR mediates K^+^ secretion into the xylem sap for translocation to shoots (21). With regard to the latter function, however, the mechanisms that enable and coordinate sustained release of K^+^ mediated by SKOR, particularly those allowing K^+^ reloading of K^+^ secreting cells, remain poorly understood.

The Shaker family comprises 9 members in *Arabidopsis* and 10 members in rice. The “tenth” member in rice is structurally distinct due to its reduced length and has no clear molecular equivalent in *Arabidopsis*. It is designated OsKAT1 (LOC_Os1g55200) in the rice genome database. Previous studies reported that OsKAT1 confers NaCl stress tolerance when overexpressed in yeast strain G19 and cultured rice cells and suggested its involvement in K^+^ uptake (44), but the functional characteristics and precise role of this short channel have not been characterized.

Here, we demonstrate that OsKAT1 is predominantly expressed in the root stele and is responsible for K^+^ uptake into stele cells, which constitutes a critical step for root-to-shoot K^+^ translocation. Functional analyses in *Xenopus* oocytes and *Arabidopsis* roots indicate that its structural shortness confers higher K^+^ uptake and transport efficiency compared to C terminus complemented “full-length” chimera, especially in the range of weakly negative membrane potentials. Surprisingly, given its inward-rectifying nature, OsKAT1 activity strongly favors K^+^ secretion into the xylem vasculature for translocation to shoots, a function previously attributed solely to outward-rectifying channels. Under field conditions, OsKAT1 function significantly contributes to grain yield in rice, highlighting its agronomic importance.

## Results

### OsKAT1 Is Primarily Expressed in Stelar Tissues of Rice Root and Is Involved in Root–Shoot K^+^ Translocation.

**Fig. 1.**
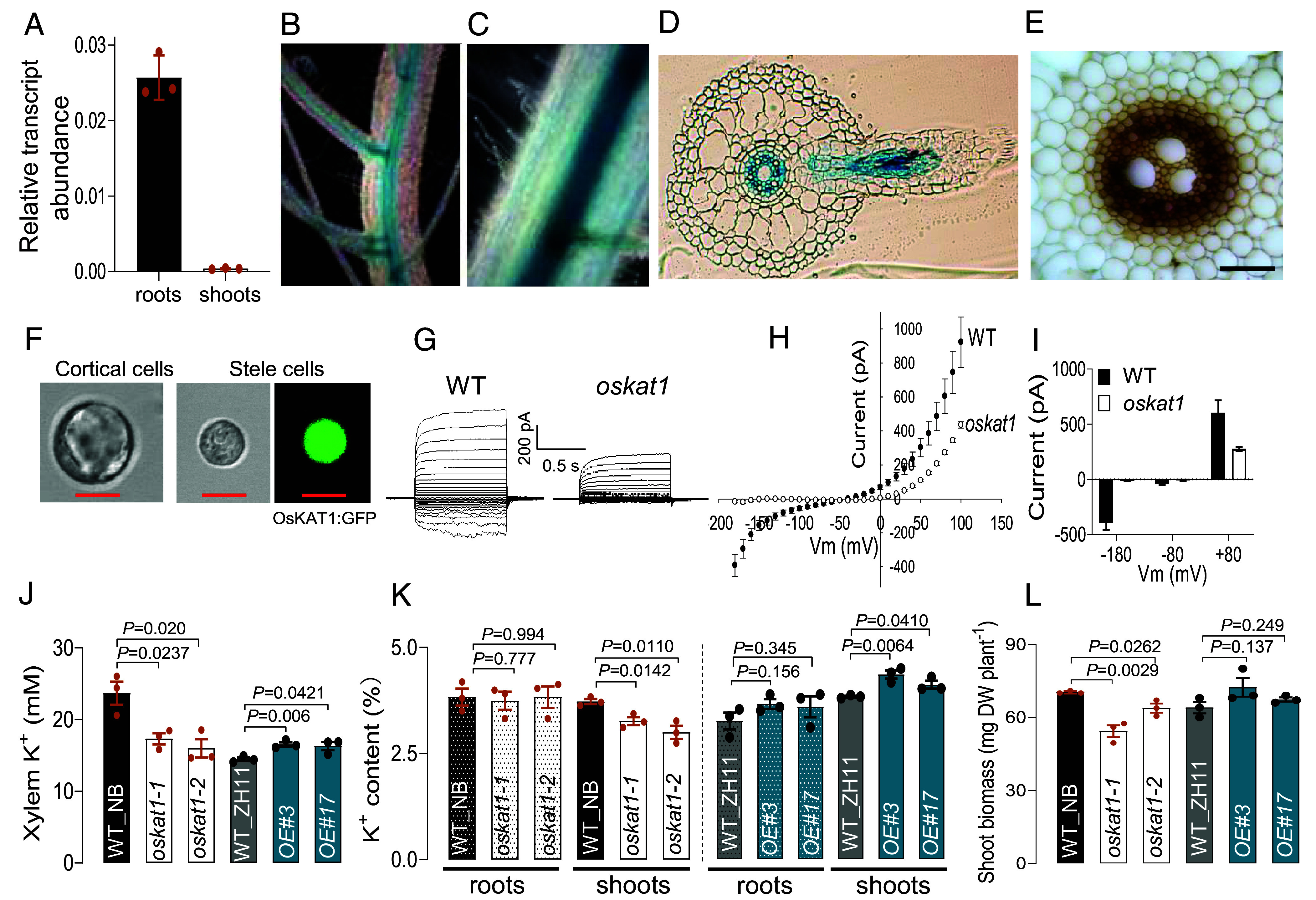
OsKAT1 is predominantly expressed in rice root stele and is involved in root–shoot K^+^ translocation. (*A*) Expression analysis of *OsKAT1* in 10-d-old rice seedlings by quantitative RT-PCR. Transcript abundance was normalized to *OsActin* (12). Data are means ± SE from three independent experiments. (*B* and *C*) Histochemical staining of GUS activity driven by the *OsKAT1* promoter. Representative images show GUS staining in the central cylinder of a seminal root (*B*) and a lateral root (*C*). (*D*) GUS staining in the cross-sections of the lateral root. (*E*) Immuno-localization of the OsKAT1-eGFP fusion protein expressed under the *OsKAT1* promoter. The fusion protein was detected using an anti-GFP primary antibody and an HRP-conjugated anti-rabbit secondary antibody, followed by visualization with DAB staining (brown deposits). (Scale bar, 50 µm.) (*F–I*) Patch-clamp recordings of K^+^ currents in stele cell protoplasts of rice roots. Stele-enriched protoplasts were isolated from the roots of 7-d-old rice seedlings and used for patch-clamp analysis. (*F*) Stele cells were distinguished from cortical cells based on their significantly smaller size and further confirmed by GFP fluorescence in protoplasts derived from transgenic rice expressing the OsKAT1:GFP fusion construct under its native promoter (*pOsKAT1*). (Scale bar, 10 μm.) (*G*) Whole-cell K^+^ currents recorded in stele cell protoplasts of WT and *oskat1* mutant roots under 10 mM external K^+^ (pH 5.8). Currents were elicited by 1.5-s voltage steps from –180 to +100 mV in 10 mV increments. A long holding at –40 mV for 1.5 s was applied to ensure complete deactivation of outward currents. (*H*) I–V relationships of whole-cell recordings in WT and oskat1 mutant cells. (*I*) Statistical summary of current amplitudes measured at membrane potentials of –180, –80, and +80 mV. Data represent means ± SE (n = 8 for WT, n = 4 for *oskat1* mutant). (*J*) K^+^ concentration (mM) in xylem sap. Xylem sap was collected from two independent *oskat1* knockout mutants (*oskat1-1* and *oskat1-2*) in the *japonica* Nipponbare background (NB), the corresponding WT controls (WT_NB), two *OsKAT1-* overexpressing lines (*OE#3* and *OE#17*) in the Zhonghua-11 background, and their corresponding WT controls (WT_ZH11). Fourteen-day-old seedlings were subjected to 3 d of K^+^ deprivation followed by 3 h of 20 mM K^+^ resupply before sap collection. All plants showed comparable visual phenotypes and biomass under applied conditions of preculture. (*K* and *L*) Ten-day-old seedlings were grown hydroponically for an additional 7 d in 10 mM K^+^ medium before sampling for K^+^ contents and biomass measurements. (*K*) Root and shoot K^+^ contents in mutants (*Left*) and the *OE* plants (*Right*), compared to their respective wild types. (*L*) Shoot biomass of mutants and the OE plants. Data are presented as means ± SE (n = 3). *P*-values were calculated using a two-tailed Student’s *t* test.

Our qRT-PCR analysis revealed that *OsKAT1* is predominantly expressed in rice roots (Fig. 1*A*). Promoter-reporter gene activity assays using *pOsKAT1*:*GUS* showed strong GUS activity specifically localized to the steles of both seminal (Fig. 1*B*) and lateral (Fig. 1*C*) roots, which was further confirmed in cross-sections of mature roots (Fig. 1*D*). Consistent with this, stable transgenic rice plants expressing OsKAT1:eGFP under the control of its native promoter *pOsKAT1* exhibited green fluorescence signals specifically in the root stele (*SI Appendix,* Fig. S1 *A* and *B*). Immunohistochemical staining with an anti-GFP antibody also confirmed the stele-specific localization of OsKAT1 (Fig. 1*E*). Further evidence for the expression and activity of OsKAT1 in stelar cells was obtained through patch-clamp recordings. In stele-enriched protoplast preparations, stele cells were distinguished from cortical cells based on their significantly smaller size (17, 18). In protoplasts derived from transgenic rice roots expressing OsKAT1:GFP under the control of native *pOsKAT1* promoter, GFP fluorescence was exclusively observed in stele cells (Fig. 1*F*), consistent with the root stele-specific localization shown in Fig. 1 *A*–*E*. At a physiologically relevant external K^+^ concentration (10 mM), significant outward and inward currents were recorded in wild-type (WT) stele cells (Fig. 1 *G*–*I*). The current–voltage (I–V) relationship indicated that the membrane remained permeable to K^+^ in the whole range of voltages, including at voltage close to the K^+^ equilibrium potential E_K._ In contrast, the absence of *OsKAT1* expression in *oskat1* KO mutant plants resulted in total suppression of the slowly activating inward current and also reduced the outward current (Fig. 1 *G*–*I*).

To investigate the physiological role of OsKAT1 in the plant, we generated *oskat1* knockout mutant (KO) and overexpression (*OE*) rice lines and selected two individual homozygous lines of each type for the analyses (*SI Appendix,* Fig. S2 *A* and *B*). In hydroponically grown seedlings, xylem sap K^+^ concentration decreased by approximately 30% in the KO mutants and increased by about 13% in the *OE* plants compared to the corresponding WT control plants (Fig. 1*J*), indicating that OsKAT1 facilitates K^+^ loading into the xylem for root-to-shoot translocation. After 7 d under sufficient K^+^ supply (10 mM), the shoot K^+^ contents were lower by 15 to 20% in the KO plants compared with the control WT plants, while the root K^+^ contents remained unchanged (Fig. 1*K*), supporting a role of OsKAT1 in K^+^ export from roots rather than uptake from the external medium. *OE* lines accumulated more K^+^ in shoots (Fig. 1*K*). Finally, the mutant plants were found to display a significant decrease in shoot biomass by 22% and 16% in the two KO lines (Fig. 1*L*). Together, these results indicate that OsKAT1 is a key player in long-distance K^+^ translocation from roots to shoots in rice, and that its absence significantly impacts plant growth.

### OsKAT1 Belongs to a Particular Type of Short Shaker K^+^ Channel.

The deduced polypeptide of OsKAT1 consists of only 502 amino acids. It displays the core structure of a Shaker K^+^ channel, but lacks the C-terminal sequences immediately downstream of the putative cyclic nucleotide-binding domain (cNBD). Sequence alignment reveals that the C terminus of OsKAT1 is approximately 170 amino acids shorter than that of AtKAT1 (KAT1-type continuation of the C-terminal tail, which contains a K_HA_ domain but no ankyrin repeats, hereafter referred to as “C1”), and about 400 amino acids shorter than OsAKT1 (AKT1-type continuation of the C-terminal tail, which contains both an ankyrin and K_HA_ domains, designated “C2”) (*SI Appendix,* Fig. S3*A*). The full-length nature of this short polypeptide was confirmed by high-fidelity PCR amplification, chromosome walking, and sequencing (*SI Appendix,* Fig. S3 *B* and *C*), supporting that OsKAT1 is an innate short Shaker K^+^ channel.

OsKAT1 shares the highest overall amino acid sequence identity with the *Arabidopsis* AtKAT1 (65%) (45) and other KAT-type channels and belongs to the subfamily #2 (28) of the five Shaker subfamilies in plants (5). In silico searches with BLASTP (expect threshold of 1e-30) (46) among annotated *Viridiplantae* genomes (NCBI refseq_protein database; September 2021) allowed the retrieval of a pool of 1,920 candidate sequences of OsKAT1 homologs. They were filtered using Prosite (47) and CD-Search (48) to retain only full-length sequences containing the essential transmembrane hydrophobic core (cl37996; Ion_Trans domain) and an intact cNBD (PS50042 domain; cNMP binding domain) that are required for a functional channel protein (49). The sequences containing either an ANK domain (PS50088 or PS50271) or a K_HA_ domain (PS51490) downstream of the cNBD were then excluded, resulting in a total of 24 independent sequences potentially coding for short Shaker channels of approximately 498 to 535 amino acids, similar to OsKAT1 (*SI Appendix,* Fig. S4*A*). Phylogenetic analysis of these 24 sequences alongside the nine *Arabidopsis* Shakers revealed that 16 out of 24 encode the short Shakers—lacking both ANK and K_HA_ domains -belong to subfamily #2 (denoted “K2” in *SI Appendix F*ig. S4*A*). It should be noted that all these short channels are from monocot species (*SI Appendix*, Fig. S4 *A* and *B*). Further analyses revealed that, among the 12 monocot species harboring at least one subfamily #2 short channel, 11 belong to *Poaceae*. These *Poaceae* short channels, along with an additional ortholog identified in *Hordeum vulgare* (HvK2.2), form a unique phylogenetic group here named GR 2.2 in reference to OsKAT1 which is also named OsK2.2 according to phylogenetic classification (*SI Appendix,* Fig. S4 *A* and *B*). Each *Poaceae* genome encodes only one such short channel. The other two groups in subfamily #2 of the *Poaceae*, GR 2.1 and GR 2.3 (*SI Appendix*, Fig. S4*B*), consist of channels with classical C-terminal regions containing a K_HA_ domain.

### Functional Characterization of OsKAT1 in *Xenopus* Oocytes.

**Fig. 2.**
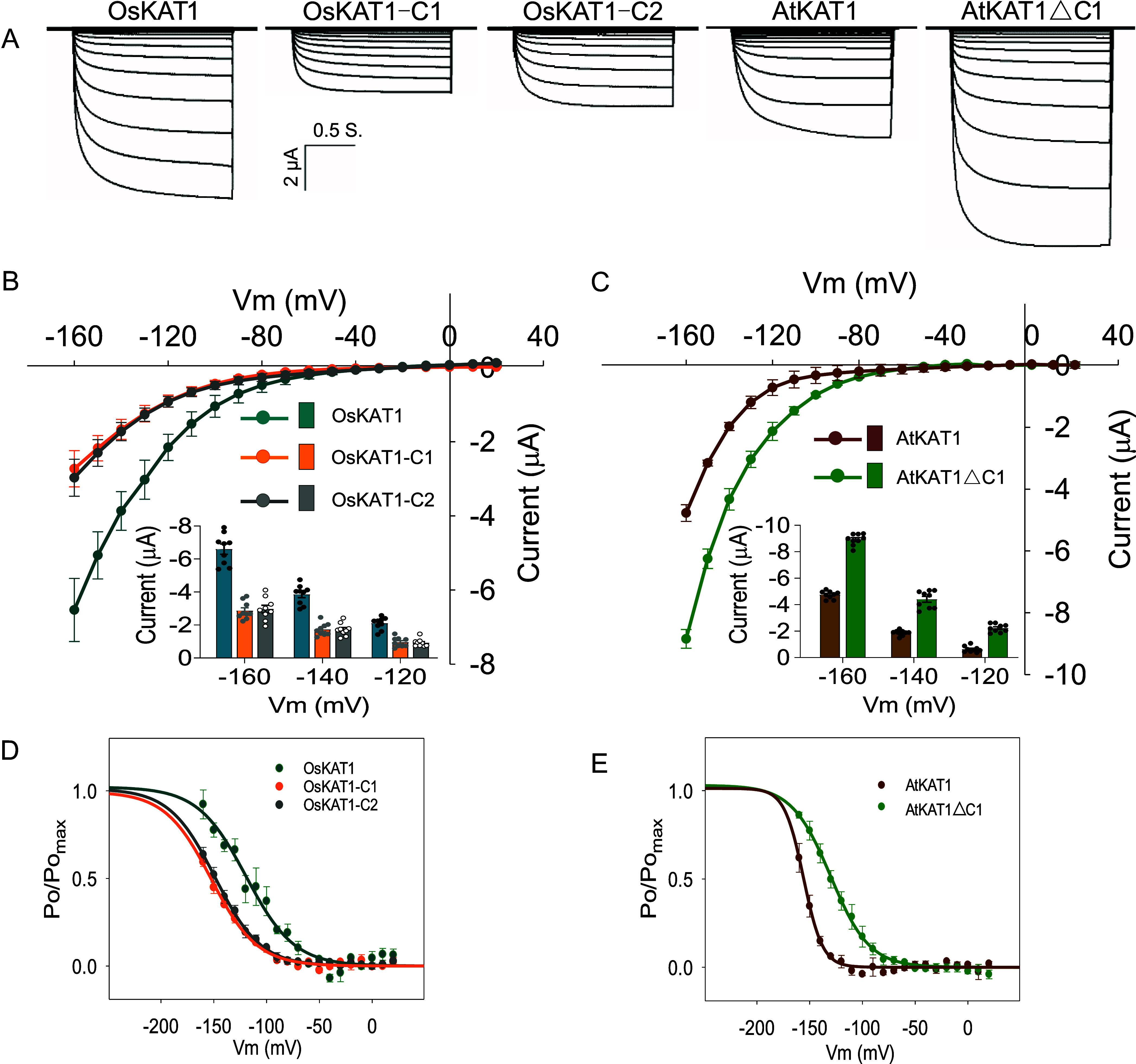
Functional characterization of OsKAT1 and its C-terminal shortness in *Xenopus* oocytes. (*A*) Representative recordings obtained in an oocyte expressing either OsKAT1, or a chimeric construct fusing OsKAT1 to either the C-terminal region C1 from AtKAT1 or C2 from OsAKT1 (OsKAT1-C1 and OsKAT1-C2, respectively), or AtKAT1 or AtKAT1 deleted from its C1 region (AtKAT1∆C1). The recording protocol was composed of 1.5-s voltage steps ranging from −160 to +20 mV with 10 mV increments (holding potential: −40 mV). The external solution bathing the oocytes contained 50 mM K^+^, pH7.4. (*B* and *C*) Current–voltage relationships of the WT OsKAT1 and of the chimeric channels OsKAT1-C1 and OsKAT1-C2 (*B*), and of AtKAT1 and the related shortened channel AtKAT1∆C1 (*C*). *Inset* bar graphs: statistical analysis of the current amplitudes at −160 mV, −140 mV, and −120 mV, respectively. Means ± SE (n = 9). (*D* and *E*) Gating properties. Voltage dependence of the relative open probability (*P_o_/P_o,max_*) of OsKAT1 and of the related chimeric channels OsKAT1-C1 and OsKAT1-C2 (*D*), and of AtKAT1 and the related shortened channel AtKAT1∆C1 (*E*). Solid lines are Boltzmann fits to the data [as previously described (25, 41)]. Means ± SE (n = 3 to 6). Means ± SE (n = 4). *P* values were derived from statistical analyses using a two-tailed Student’s *t* test.

Electrophysiological analyses using the two-electrode voltage-clamp technique in *Xenopus* oocytes were carried out to investigate the functional characteristics of OsKAT1. Furthermore, two chimeric constructs—OsKAT1-C1 and OsKAT1-C2 (*SI Appendix,* Fig. S3*A*)—were generated and comparatively expressed in oocytes to examine the consequences of the absence of a conventional C terminus (i.e., the absence of either a KAT1- or AKT1-type C terminus). Electrophysiological recordings indicated that OsKAT1 is fully functional despite its short C terminus, and gives rise to a bona fide voltage-gated slowly activating inward-rectifying K^+^ conductance, reminiscent of the behaviors of AtKAT1 (50) and OsAKT1 (12) (Fig. 2 *A*, *Left* panel; Fig. 2*B*).

Both chimeric channels, OsKAT1-C1 and OsKAT1-C2, also functioned as inward rectifiers. However, the presence of either C1 or C2 resulted in 50-60% reduction in inward K^+^ currents compared to WT OsKAT1 (Fig. 2 *A* and *B*). Conversely, reciprocal deletion of the corresponding C1 segment in AtKAT1 (AtKAT1∆C1; *SI Appendix,* Fig. S3*A*) increased current amplitudes by 90 to 120% relative to WT AtKAT1 (Fig. 2 *A*–*C*). These results were consistently reproduced across multiple independent experiments, with at least 50 oocytes tested per construct. Statistical analysis of pairwise comparisons (Fig. 2 *B* and *C*) supports the conclusion that the C1 and C2 domains intrinsically reduce macroscopic current amplitude. Voltage sensitivity analyses revealed that attaching of C1 or C2 to OsKAT1 shifted the channel activation curve toward more negative voltages, with a half-activation potential change of approximately −30 mV (Fig. 2*D*). In contrast, removing the C1 domain from AtKAT1 shifted the half-activation potential by about +30 mV (Fig. 2*E*). No significant differences were observed between OsKAT1 and OsKAT1-C1 in other functional properties, including sensitivity to external K^+^ concentration, pH regulation, Ba^2+^ blockade, or ionic selectivity (*SI Appendix*, Fig. S5 *A–E*). Furthermore, short channels without a C terminus (OsKAT1 and AtKAT1∆C1) exhibited faster activation kinetics, as indicated by a roughly 50% reduction in half-activation time constants compared to their full-length counterparts (*SI Appendix*, Fig. S5*F*).

Collectively, the whole set of electrophysiological data indicate that the short channel OsKAT1 activates at less hyperpolarized membrane potentials than AtKAT1. Such a shift in activation potential results in higher K^+^ uptake activity under the same voltage conditions. From a thermodynamic perspective, a positive shift in activation potential of an inward-rectifying channel could potentially increase K^+^ leakage/efflux by expanding the voltage range in which the channel remains open at voltages positive to the K^+^ equilibrium potential (*Ek*). However, current–voltage relationships recorded between –140 and +20 mV showed no significant K^+^ efflux at voltages positive to the inward current threshold (see bath conditions with 1 and 5 mM K^+^ in *SI Appendix*, Fig. S5*A*, where the activation potential of OsKAT1 is less negative than the *Ek*), indicating that OsKAT1 is primarily dedicated to mediating K^+^ influx and is unlikely to contribute significantly to K^+^ leakage or efflux.

### Molecular Dynamics Simulations.

**Fig. 3.**
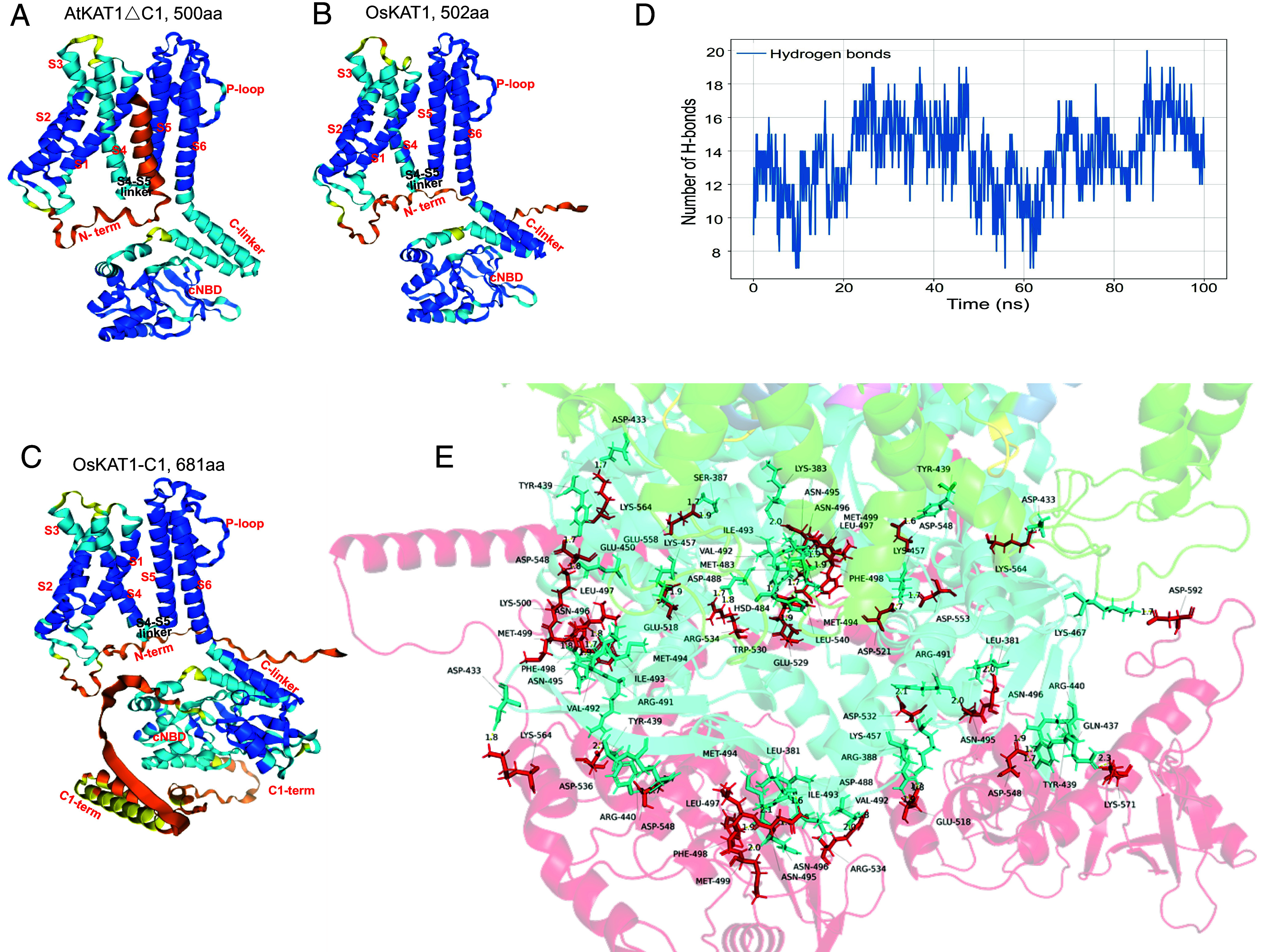
Structure prediction and molecular dynamics simulation. (*A* and *B*) The AlphaFold3.0 structures of AtKAT1∆C1 (Q39128) and OsKAT1 (Q5JM04) were predicted according to the structures provided in the AlphaFold Protein Structure Database (https://alphafold.ebi.ac.uk/) that neglects the distal C terminus (C1-term) in the AtKAT1 structure. AtKAT1∆C1 (*A*) and OsKAT1 (*B*) structures were annotated based on previous Cryo-EM resolution of the *Arabidopsis* AtKAT1 structure (51, 52). (*C*) The elongated chimera channel OsKAT1-C1 structure that contained a C1 terminus (C1-term) obtained from AtKAT1 was built using AlphaFold3.0. Transmembrane helices S1–S6 and the P-loop, S4–S5 linker, C-linker, and the C1-term were indicated to each single subunit structures. Helices highlighted in deep blue, light blue, yellow, and orange respectively represent very high, high, low, and very low modeling confidence with pLDDT >90%, 70 to 90%, 50 to 70%, and <50%, respectively. (*D* and *E*) Molecular dynamics simulations were performed in 150 mM KCl environment to predict possible interactions between the C1-term and the C-linker. The presence of Hydrogen (H) bonds interactions was simulated for a time course of 100 ns using GROMACS (*D*) and visualized at 100 ns on the tetramer structure of OsKAT1-C1 (*E*). The structures of the C-linker (cyan) and the C1-term (red) were highlighted.

To gain structural insights into the activation mechanism of the short-channel OsKAT1, we performed molecular dynamics simulations using GROMACS. The core structure of OsKAT1 (UniProt: Q5JM04), as predicted in the AlphaFold Protein Structure Database (https://alphafold.ebi.ac.uk), is nearly identical to that of the *Arabidopsis* AtKAT1 (UniProt: Q39128) (Fig. 3 *A* and *B*). Our simulations yielded predictions consistent with previous cryo-EM structures of AtKAT1 (51, 52), indicating that in OsKAT1, the C-linker motif (the first domain downstream of the channel hydrophobic core) serves as a key structural element interacting with both the fourth (S4, the so-called voltage-sensor) and the sixth (S6) transmembrane segments of the channel transmembrane core via respectively, 7 to 14 and 18 to 30 hydrogen bonds (*SI* *Appendix*, Fig. S6). These results suggest that the activation model proposed for AtKAT1 (51, 52)—which does not include its distal C terminus (52)—may also apply to the short-channel OsKAT1 (*SI* *Appendix,* Fig. S7*A*). We subsequently constructed a 681-amino-acid full-length model, designated OsKAT1–C1, by extending OsKAT1 with the C1 terminus from AtKAT1 (Fig. 3*C*). Stable interactions involving 7 to 20 hydrogen bonds were predicted between the fused C1 terminus and the C-linker (Fig. 3 *D* and *E* and Movies S1 and S2), suggesting that, in addition to the existing model, the C terminus contributes to force transmission in the elongated OsKAT1–C1 through interactions with the C-linker; whereas virtually no hydrogen bond interaction was predicted between the C1 terminus and either S4 or S6. We also found that the intracytoplasmic S4–S5 linker forms 15 to 22 hydrogen bonds with S4, and 4 to 9 hydrogen bonds with S6. This finding supports the existing model (52) in which the S4–S5 linker participates in the force transmission during the S4 movements triggered by changes in transmembrane polarization. The additional interactions between the C1 terminus and the C-linker are predicted to become part of the force–transmission complex, thereby increasing its structural complexity. As a result, the full-length channel, which retains the C terminus, requires a larger S4 displacement driving force and a stronger transmembrane polarization to open the gate (*SI* *Appendix,* Fig. S7*B*). In contrast, the short channel OsKAT1, which lacks the C terminus, exhibits reduced structural complexity and consequently opens with less S4 displacement under a weaker transmembrane hyperpolarization (*SI* *Appendix,* Fig. S7*C*). These predicted interactions provide a theoretical basis for explaining the facilitated opening observed in the short channels OsKAT1 and AtKAT1∆C1 at lower levels of membrane hyperpolarization, as revealed by electrophysiological analyses (Fig. 2). Furthermore, they also allow to explain the faster activation kinetics and reduced half-activation time (t_1/2_) observed in the short channels (*SI Appendix*, Fig. S5*F*).

In rice, the OsAKT1 and OsAKT2 channels, which contain ANK domains within their C2-type terminus, are activated by the Ca^2+^-sensing kinase complex OsCBL1/OsCIPK23 (12, 27) in a similar way as the *Arabidopsis* AtAKT1 (53). Neither OsKAT1 nor the chimeric OsKAT1-C1 exhibited regulation by the OsCBL1/OsCIPK23 complex when the proteins were coexpressed in oocytes (*SI Appendix,* Fig. S8 *A* and *B*). In contrast, constructs containing ANK domains, namely OsKAT1-C2 and OsKAT1-C1-ANK (a chimera of OsKAT1-C1 comprising an inserted ANK domain obtained from OsAKT1; see *SI Appendix,* Fig. S3*A*), were strongly activated by the complex, showing an approximately 50% increase in K^+^ uptake current (*SI Appendix,* Fig. S8 *A*–*D*). These results demonstrate that the presence of an ANK domain, whether native or heterologously introduced, is necessary and sufficient for modulation by the CBL/CIPK complex (54, 55). Activation of OsKAT1-C1-ANK and OsKAT1-C2 by OsCBL1/OsCIPK23 also shifted their gating kinetics, moving the half-activation potential by approximately +30 mV (*SI Appendix,* Fig. S8*E*).

### Functional Expression in *Arabidopsis*.

**Fig. 4.**
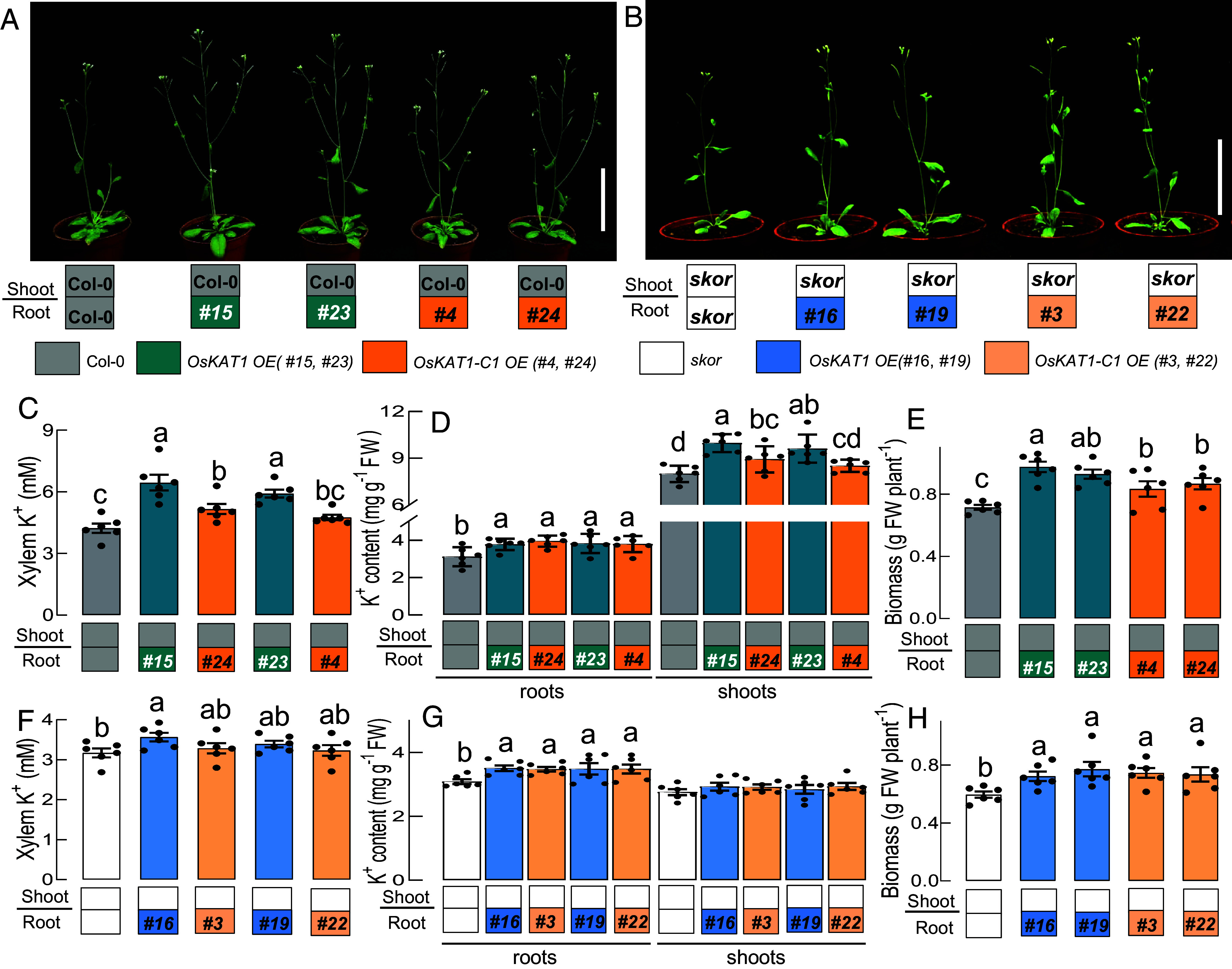
Functional characterization of OsKAT1 and its elongated chimera OsKAT1-C1 in grafted *Arabidopsis* transgenic plants. Grafted seedlings were constructed using different genetic backgrounds. The gray box denotes scions or rootstocks from Col-0; aqua green indicates rootstocks from OsKAT1 overexpression (OE) lines (#15, #23) in Col-0; deep orange indicates rootstocks from OsKAT1-C1 OE lines (#4, #24) in Col-0; white represents scions or rootstocks from *skor* mutant; blue represents rootstocks from OsKAT1 OE lines (#16, #19) in *skor* mutant background; light orange indicates rootstocks from OsKAT1-C1 OE lines (#3, #22) in *skor* background. Graft combinations were shown below each panel. (*A* and *B*) representative images of 4-wk-old grafted plants expressing OsKAT1 or OsKAT1-C1 in rootstocks, with scions from Col-0 (*A*) or *skor* mutants (*B*). (Scale bar, 10 cm.) (*C*) K^+^ concentration (mM) in xylem sap samples from grafted plants with Col-0 rootstocks expressing OsKAT1 or OsKAT1-C1 and Col-0 scions. (*D* and *E*) Root and shoot K^+^ contents (*D*) and fresh shoot biomass (*E*) of grafted plants with Col-0 rootstocks expressing the transgenes and Col-0 scions. (*F*) K^+^ concentration (mM) in xylem sap from grafted plants with *skor* rootstocks expressing OsKAT1 or OsKAT1-C1 and *skor* scions. (*G* and *H*) Root and shoot K^+^ contents (*G*) and fresh shoot biomass (*H*) of grafted plants with *skor* mutant rootstocks expressing the transgenes and *skor* scions. Data are presented as means ± SE (n = 6). Different letters indicate statistical significances at *P* < 0.05 (one-way ANOVA with Duncan’s multiple-comparison test).

To further elucidate the *in planta* function of OsKAT1, OsKAT1, and its elongated chimera OsKAT1-C1 were expressed in *Arabidopsis,* in both the WT (Col-0) and the *skor* mutant backgrounds. Homozygous T3 transgenic seedlings were grown on agar plates and transgene expression levels were confirmed by PCR for each independent line. Two paired lines showing comparable expression of OsKAT1 or OsKAT1-C1 in each genetic background were selected for the analyses (*SI Appendix,* Fig. S9). Through grafting, transgene expression was confined solely to the root system. Successful grafts were grown for 4 weeks under conditions of 1 mM K^+^ for phenotypic analyses (Fig. 4 *A* and *B*).

In the Col-0 background, where the outward rectifier AtSKOR is functional, both OsKAT1 and OsKAT1-C1 expression significantly increased K^+^ concentrations in the xylem sap collected over 3 h (Fig. 4*C*). Interestingly, the OsKAT1 lines displayed a stronger increase than the OsKAT1-C1 lines: Compared with the control WT grafted plants, the two *OsKAT1* lines (#15 and #23) showed *ca.* 50% and 39% increases, respectively, while the two *OsKAT1-C1* lines (#24 and #4) displayed 22% and 12% increases. After 4 wk of growth, these differences in xylem K^+^ transport led to altered K^+^ contents in the shoots, while the root K^+^ levels were only slightly affected. OsKAT1 expression in roots increased the shoot K^+^ contents by 20 to 25%, whereas OsKAT1-C1 expression only led to an increase of 6 to 12%, relative to the control WT grafted plants (Fig. 4*D*). Consistent with improved K^+^ allocation to shoots, OsKAT1-expressing plants also produced more shoot biomass:increases of 30 to 36% were observed in OsKAT1 lines, but only of 16 to 21% in OsKAT1-C1 lines (Fig. 4*E*). These results indicate that root-specific expression of both OsKAT1 and OsKAT1-C1 enhances K^+^ translocation via the xylem, promotes shoot K^+^ accumulation, and stimulates plant growth. Whereas these beneficial effects are more pronounced with OsKAT1 than with OsKAT1-C1.

In the *skor* mutant background, which lacks AtSKOR-mediated K^+^ secretion activity in the stele, both OsKAT1 and OsKAT1-C1 expression resulted in less than 10% increase in xylem sap K^+^ concentration, with no clear difference between the two channel types (Fig. 4*F*). Accordingly, shoot K^+^ content remained low and comparable to the control grafts, despite significant K^+^ accumulation in roots (Fig. 4*G*). Nonetheless, some growth improvement was still detectable in the transgenic *skor* plants (Fig. 4*H*).

### OsKAT1 Overexpression Enhances Salt Tolerance in Rice.

To further evaluate the role of OsKAT1 in salt stress response, hydroponically grown OsKAT1 mutant and OE rice seedlings (10-d-old) were subjected to 100 mM NaCl treatment for 14 d. Compared to their corresponding WT controls, the *oskat1* mutants showed a reduction of approximately 25% in shoot biomass, while an increase of around 25% in shoot biomass was observed in the *OE* plants. These phenotypic changes were associated with an altered K^+^/Na^+^ homeostasis in the shoots of the mutant and *OE* lines (*SI Appendix,* Fig. S10). These whole-plant results are consistent with previous observations in yeast and rice *calli* cells (44), allowing to conclude that overexpression of OsKAT1 promotes plant K^+^ accumulation and reduces Na^+^ net uptake, thereby enhancing salt tolerance in rice.

### OsKAT1 Expression Strongly Contributes to Rice Grain Yield Under Field Conditions.

**Fig. 5.**
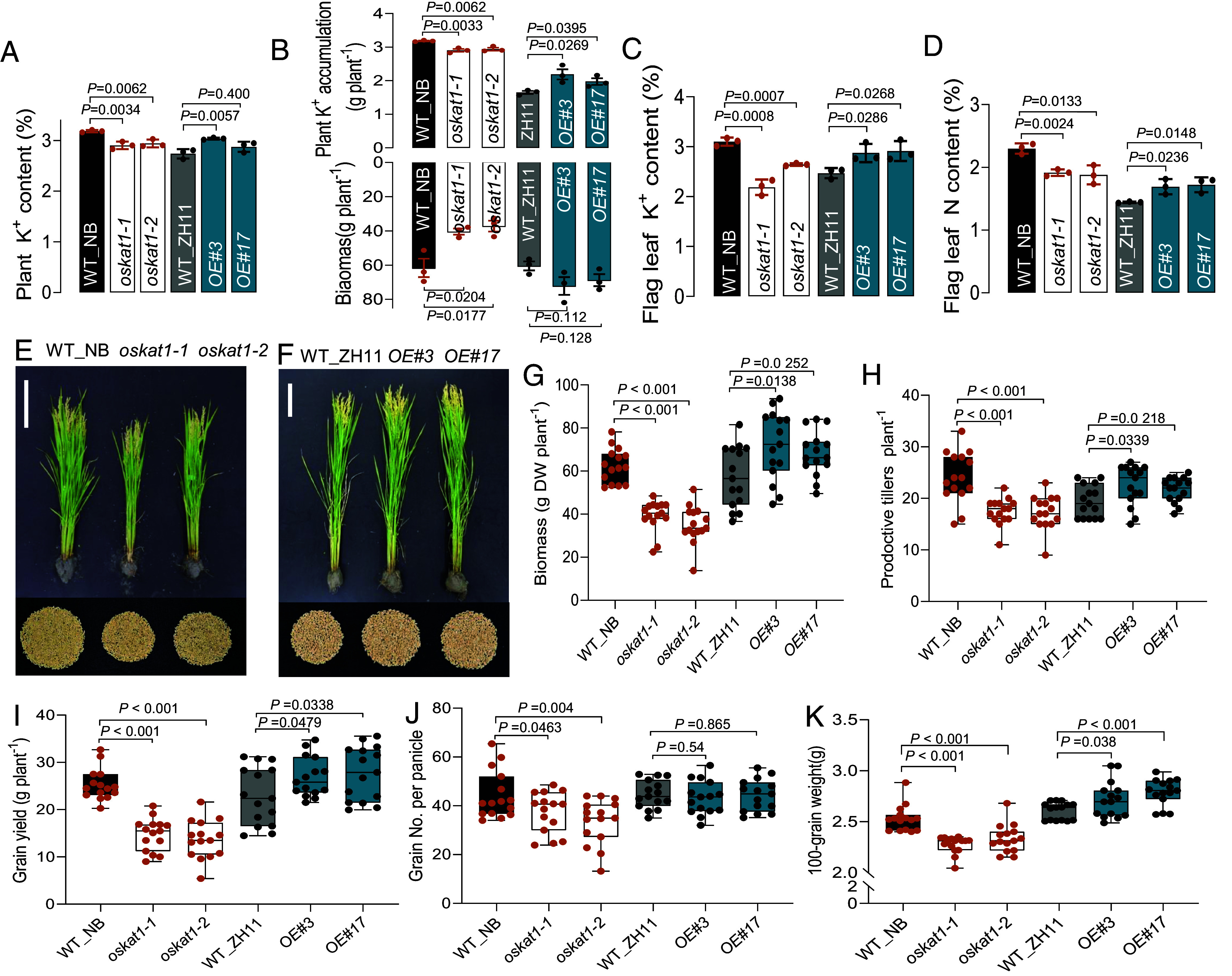
OsKAT1 significantly contributes to rice grain yield under field conditions. WT (Nipponbare and ZH11), *oskat1* knockout mutants (*oskat1-1, oskat1-2*), and OsKAT1 overexpressors (*OE#3, OE#17*) were grown under field conditions with NPK fertilization (200 kg N, 90 kg P_2_O_5_ and 240 kg K_2_O per hectare). (*A*–*D*) Measurements from 3 representative plants at the grain-filling stage (T4 generation): shoot K^+^ contents (*A*), aerial biomass and total shoot K^+^ accumulation (*B*), flag leaf K^+^ (*C*), and N (*D*) contents. Total shoot K^+^ accumulation was calculated as shoot K^+^ content multiplied by shoot dry biomass. (*E, F*) Representative images of plants at the grain-filling stage and grain yield per plant at maturity: (*E*) *oskat1* mutants versus Nipponbare wild type (WT_NB); (*F*) OsKAT1-OE lines versus ZH11 wild type (WT_ZH11). (Scale bar, 20 cm.) (*G–K*) Yield-related traits from the 2019 trial: plant dry matter (*G*), number of productive tillers per plant (*H*), grain yield per plant (*I*), grain number per panicle (*J*) and 100-grain weight (*K*). Data are presented as means ± SE (n = 15). Box plots indicate maxima, first quartile, median, third quartile, and minima. *P* values were determined using a two-tailed Student’s *t* test.

To evaluate the physiological relevance of OsKAT1 under realistic agronomic conditions, field trials were conducted during the 2017 and 2019 rice growing seasons in a typical rice paddy field. Prior to planting, the topsoil was amended with K_2_SO_4_ fertilizer (240 kg K_2_O per hectare) in order to ensure that K^+^-availability would be around the sufficient range (e.g., 150 ppm). Assays performed during the grain-filling stage revealed that *oskat1* mutant plants displayed a ~9% reduction in shoot K^+^ content and a loss of biomass greater than 30% compared to WT plants, resulting in significantly lower total shoot K^+^ accumulation (based on K^+^ content multiplied by dry weight). In contrast, the OE lines showed increased values for these traits (Fig. 5 *A* and *B*). The flag leaf K^+^ content decreased by 20 to 25% in *oskat1* mutant plants but increased by 15% in *OE* plants relative to the corresponding WT (Fig. 5*C*). Similarly, flag leaf nitrogen content was reduced by about 18% in the mutants and raised by 17% in the *OE* plants (Fig. 5*D*). These changes in K^+^ and N content in photosynthetic leaves should have significant effects on stomatal function. Consistent with this, experiments conducted on hydroponically grown plants revealed that *oskat1* mutants had reduced stomatal conductance (16 to 18%), photosynthesis (22 to 26%), and transpiration rates (28 to 32%), whereas OE lines exhibited increases of 15 to 25%, 30 to 35%, and 15 to 30% of the corresponding traits, compared to WT (*SI Appendix,* Fig. S11). These results indicate that OsKAT1 contributes to maintaining leaf K^+^ and nitrogen levels, thereby supporting stomatal activity and prolonging photosynthetic efficiency—ultimately resulting in improved rice growth and grain yield under field conditions (Fig. 5 *E*–*K* and *SI Appendix,* Fig. S12). Indeed, in the 2019 trial, *oskat1* mutants displayed severe agronomic impairments: a ~40% reduction in shoot biomass, 25 to 30% fewer productive tillers per plant, and finally a loss of about 40% in grain yield compared to WT. Conversely, OE plants showed roughly 20% increases in shoot biomass, tiller number, and grain yield (Fig. 5 *G*–*I*). The mutants also exhibited reduced grain number and lower 100-grain weight, indicating compromised grain filling, whereas OE plants showed higher 100-grain weight than the WT plants (Fig. 5 *J* and *K*). Results from the 2017 trial, which focused on plant biomass, tillering, and grain yield per plant, were consistent with those from 2019 (*SI Appendix,* Fig. S12). Taken together, these results indicate that, although OsKAT1 is predominantly expressed in roots, this channel plays a critical role in maintaining K^+^ and nitrogen homeostasis in shoots, thereby improving photosynthetic performance and ultimately increasing grain yield under field conditions.

## Discussion

### The Presence of OsKAT1-Type Short Shaker Potassium Channel Is a Common Trait Among *Poaceae* Crops.

In animals, “Shaker-type” channels that possess a cNBD are characterized as Kv10 to Kv12 channels, also known as KCNH1–8 or Eag, Erg, and Elk channels (56). These channels, which typically comprise 960 to 1,200 amino acids, contain an extended C -terminal region downstream of the cNBD and function as voltage-gated outward rectifiers. Mammalian genomes also encode shorter Shaker-like subunits, ranging from 470 to 570 amino acids. For instance, the mouse and rat genomes contain eight and three short isoforms of KCNQ1 (also referred to as Kv7.1 or KvLQT1), respectively. Although these isoforms also exhibit C-terminal “truncation,” they share limited sequence similarity with OsKAT1 and are nonfunctional when expressed alone. Instead, they interact with full-length KCNQ1 subunits and participate in the modulation of action potential repolarization in excitable cells in response to various ligands and neurotransmitters (57–60). OsKAT1 displays the conserved Shaker-type hydrophobic core and an intact cNBD, similar to all plant Shaker channels, but lacks the distal C-terminal region present downstream of the cNBD in other plant Shakers such as AtKAT1 and AtAKT1. It belongs to a class of short Shaker channels specific to monocots, particularly within *Poaceae* species. Although OsKAT1 was previously cloned through functional complementation in yeast and shown to enhance salt tolerance when overexpressed in yeast and rice cell cultures (44), its detailed channel properties and physiological role in rice plant—particularly with respect to its naturally truncated C terminus—were still not well understood. Moreover, orthologs of OsKAT1 in other cereals, such as maize and barley, have not yet been functionally characterized (*SI Appendix*, Fig. S4). Cereals generally produce higher biomass and grain yields compared to dicot crops like legumes. For instance, soybean yields approximately 5 t/ha (61); whereas rice often achieves 8 to 10 t/ha (62, 63), resulting in greater K^+^ demand in aerial tissues. Given the CO_2_-limited nature of photosynthesis in rice (64), the presence of a short Shaker channel like OsKAT1 in the root stele facilitates root-to-shoot K^+^ translocation, thereby supporting stomatal opening and photosynthetic efficiency—key traits that contribute to high productivity.

In this study, we show that OsKAT1 is very predominantly expressed in the stele of rice roots. This finding challenges the previous report based on semiquantitative RT-PCR analysis, which claimed that OsKAT1 expression was restricted to the internodes and rachises in mature rice plants and undetectable in young seedlings and their roots (44). However, our multimethodological approach—combining qRT-PCR analyses, promoter:GUS reporter assays, immunolabeling of a *pOsKAT1*:OsKAT1-eGFP fusion construct as well as patch-clamp recordings in stele cell protoplasts (Fig. 1 and *SI Appendix*, Fig. S1)—provides evidence that *OsKAT1* is primarily expressed in the root stele and furthermore, the encoded channel is active in the membrane of this cell type. To reconcile this discrepancy with the initial report, we cultivated rice seedlings (*Oryza sativa* L. cv Nipponbare) under both our conditions and those described by Obata et al. (44). Semiquantitative RT-PCR under both regimes confirmed *OsKAT1* expression “exclusively” in roots across four biological replicates (*SI Appendix*, Fig. S13*A*). However, in mature field-grown plants, we also detected expression in internodes and rachises, suggesting that *OsKAT1* expression may be developmentally regulated and extends to additional tissues (*SI Appendix*, Fig. S13*B*).

### Functional Significance of the C-Terminal Shortness in OsKAT1.

Shaker channels are tetrameric proteins. The functional protein results from the assembly of 4 Shaker polypeptides. Evidence has been obtained that the cNBD is involved in the tetramerization process (65). In addition, different regions downstream of the cNBD in AKT1- and KAT1-type Shaker channels have also been proposed as being involved in channel subunit tetramerization and/or heterotetramerization between channel subunits (65, 66). The fact that expression of OsKAT1 in *Xenopus* oocytes gives rise to functional channels indicates that the encoded polypeptide includes at least one domain that allows channel tetramerization. It can be assumed that the cNBD is one of them.

Previous studies on the *Arabidopsis* AtKAT1 channel using deletion mutants have shown that the absence of regions downstream of the cNBD alters the channel sensitivity to voltage (49). In line with these results, the present study indicates that, in OsKAT1, the innately short length of the region downstream of the channel hydrophobic core results in specific electrophysiological properties. We show that OsKAT1 transmits significantly larger currents than its elongated chimera and that this increase in current can be largely ascribed to a positive shift in the channel activation voltage, as demonstrated in oocyte experiments (Fig. 2). Molecular dynamics simulations offer further structural insight: The absence of a distal C terminus results in simpler and/or more compact force–transmission complex, reducing the electrical driving force required to open the channel gate (Fig. 3 and *SI Appendix*, Fig. S7).

Interestingly, the I–V curves provided by the patch-clamp recordings (Fig. 1 *G–I*) showing that OsKAT1 is active in stelar cells, also reveal that the membrane of these cells exhibits significant permeability to K^+^ at virtually all physiological voltages, even between −100 and −50 mV. The observation that the membrane remains significantly permeable to K^+^ in this range of physiological potentials is very different from what has been reported repeatedly in another cell type, guard cells. For instance, guard cell I/V curves in *Arabidopsis* (41), rice (22) or *Vicia faba* (67) indicate that, in this cell type, the membrane conductance to K^+^ is both very weak and poorly dependent on the voltage in this range of membrane potentials. This difference is mainly due to the fact that the activation potential of the K^+^ inward rectifiers at work in these guard cells is significantly more negative than that of OsKAT1. Thus, the short length of OsKAT1 and the resulting shift of the channel activation potential toward less negative voltages make the membrane of rice stelar cells significantly permeable to K^+^ across the entire range of physiological membrane potentials. It is reasonable to assume that this property (and the difference from guard cells) should play a fundamental role in the physiology of ion transport by rice stelar cells.

### OsKAT1 Activity Is Involved in Root–Shoot K^+^ Translocation.

The fact that OsKAT1 is significantly involved in K^+^ secretion into the xylem sap and long-distance transport of this ion to the aerial parts was initially surprising, given that only SKOR-type channels had previously been characterized as involved in this function and that OsKAT1 exhibits a strong inward rectification. Indeed, electrophysiological experiments in oocytes indicated that OsKAT1 exhibits negligible steady-state outward current at membrane voltages positive to the K^+^ equilibrium potential (Fig. 2 and *SI* *Appendix*, Fig. S5). These analyses provided evidence that OsKAT1 was unlikely to directly mediate the outward K^+^ currents that results in K^+^ secretion into the xylem sap toward the shoots. Functional analyses of OsKAT1 activity in various plant contexts, particularly when expressed specifically in *Arabidopsis* roots by grafting, clearly demonstrate that this channel alone, in absence of the stelar outward rectifier AtSKOR, does not substantially mediate upward K^+^ transport to shoots.

K^+^ ions that move up into the xylem vasculature may either have reached the stele from the cortex via the plasmodesmata that connect the cortex to the stele through the endoderm, or have been unloaded from the phloem into the root stele. There is evidence that the latter process, i.e., the recirculation of K^+^ from shoots to roots, often provides most of the ions that move up in the xylem sap toward the shoots (22, 27, 42). It has been proposed that reuptake by stelar cells of these recirculated K^+^ ions contribute to feed the secretion of K^+^ into the xylem sap (68). Due to its broad expression in the stele and its inward rectifying nature, OsKAT1 could play a major role in this “feeding” process, which would explain its significant contribution to K^+^ translocation to shoots. Patch-clamp recordings of rice stelar cells protoplasts from WT and *oskat1* KO mutant plants (Fig. 1 *G*–*I*) also reveal that the absence of OsKAT1 activity resulted in a significant reduction of the outward K^+^ currents across the membrane of these cells. This finding indicates that some coregulation of the inward and outward K^+^ conductances of the plasmalemma occurs in stelar cells, a process that also could result in indirect contribution of OsKAT1 to K^+^ translocation to shoots. Among the nine Shaker K^+^ channels in *Arabidopsis*, SKOR is the only one exhibiting a root stele-specific expression pattern. In the rice genome database, two loci—LOC_Os04g36740 and LOC_Os06g14030—have been proposed as putative functional orthologs of SKOR. Of these, LOC_Os06g14030 (designated OsK5.2) shows a broad expression pattern, notably including guard cells and the root stele, where it is involved in K^+^ secretion into the xylem sap for translocation to shoots (22, 23) like SKOR in *Arabidopsis*. The other candidate, LOC_Os04g36740, has not been successfully characterized so far.

The energetics context of ion membrane transport that allows K^+^ secretion into xylem sap is still unknown. One hypothesis proposes oscillatory membrane potentials in xylem parenchyma cells, alternating between hyperpolarized and depolarized states (26). In this model, hyperpolarization phases involve H^+^-ATPase activity, resulting in inward K^+^ channels operation, leading to K^+^ accumulation in these cells, while depolarization phases involve anion channels and other depolarizing factors that activate SKOR-like channels to secrete K^+^ into the xylem sap. In this oscillatory context, activation of inward K^+^ channels like OsKAT1 would tend to depolarize the membrane and to exit the hyperpolarized state if the incoming positive charge is not fully counterbalanced (e.g., by H^+^ extrusion). Conversely, SKOR-mediated K^+^ secretion would tend to repolarize the membrane, thereby sustaining membrane potential oscillations. Since the activation potential of OsKAT1 is only weakly negative (compared to that of other inward rectifiers such as AtKAT1), this channel as well as SKOR-like stelar outward rectifiers can remain open within a broad range of membrane voltages (as demonstrated by the patch-clamp recordings: Fig. 1 *G*–*I*), a state of the membrane permeability to K^+^ that shifts the membrane potential toward the K^+^ equilibrium potential *E_K_*. Oscillations of the membrane potential around *E_K_* would underlie the secretion of K^+^ within the xylem sap (and probably ensure a certain degree of coordination with long-distance transport of nutritive anions). Clearly, the energetics context and the potential functional coupling between OsKAT1 and SKOR-like channels in the stele of rice roots raise highly interesting questions that will deserve further investigation.

In summary, we have identified a particular type of K^+^ inward rectifying channel in rice, OsKAT1, which has no equivalent in *Arabidopsis* or other dicots due to the shortness of the sequence downstream of the channel hydrophobic core, a structural feature that results in a weakly negative activation potential. This channel mediates inward currents and is expressed in the root stele. In *Arabidopsis*, xylem loading mediated by the outward K^+^ channel SKOR in the root stele is well documented as a key mechanism for root-to-shoot K^+^ translocation (1, 9, 21). Similarly in rice, the outward Shaker channel OsK5.2 has been shown to be involved in this process (22, 23). The observation that the phenotypic defects displayed by *oskat1* KO mutant plants are analogous to those described in *atskor* and *osk5.2* KO plants, despite the inward rectifying nature of OsKAT1, reveals that this channel contributes to a previously unrecognized component of K^+^ translocation from roots to shoots, which is furthermore found to play a significant role in rice adaptation to environmental conditions as well as in plant growth and grain yield under field conditions.

## Materials and Methods

Information on plant materials used, growth conditions, and experimental methods employed in this study is detailed in *SI Appendix*. The methods include the specifics related to vector construction and plant transformation, gene expression, (immune)-histochemical staining, electrophysiological measurements and analysis, phylogenetic analysis, plant treatment, root-specific channel gene expression in *Arabidopsis* by grafting, phenotype analysis, agronomic trait evaluation, and ion content determination. Primers used in this study are listed in *SI Appendix,* Table S1. Accession references for phylogenetic classification are listed in *SI Appendix*, Table S2.

## Supplementary Material

Appendix 01 (PDF)

Movie S1. Dynamic interactions between a classical C1-terminus and the C-linker were predicted with molecular dynamics simulation using Gromacs. 8-second movies were captured at time step of 100ns. Movie 1: front view; Movie 2: end view. Red: C1-terminus; Cyan: C-linker; Violet: S6 helix; Sky blue: S4 helix; Yellow: S4-S5 linker; S1, S2, S3 and S5 helices were shown in green.

Movie S2. Dynamic interactions between a classical C1-terminus and the C-linker were predicted with molecular dynamics simulation using Gromacs. 8-second movies were captured at time step of 100ns. Movie 1: front view; Movie 2: end view. Red: C1-terminus; Cyan: C-linker; Violet: S6 helix; Sky blue: S4 helix; Yellow: S4-S5 linker; S1, S2, S3 and S5 helices were shown in green.

## Data Availability

All datasets are available at https://doi.org/10.6084/m9.figshare.31062469 (69). All study data are included in the article and/or supporting information.
