# Supplementary material for: OsKAT1 is a short Shaker potassium channel involved in root-to-shoot potassium translocation and contributes to rice grain yield
Source: Proc Natl Acad Sci U S A. 2026 Jan 28;123(5):e2527650123. doi: 10.1073/pnas.2527650123 (PMC12867649; doi:10.1073/pnas.2527650123)
Supplement: Supplementary file 1 — Appendix 01 (PDF) [file pnas.2527650123.sapp.pdf]

Supporting Information for

OsKAT1 is a short Shaker potassium channel involved in root to shoot potassium translocation and contributes to rice grain yield

Shunying Yang[a], Thanh-Hao Nguyen[b,1], Cécile Fizames[b], Junlin Li[c], Sheliang Wang[d], Aurore Vernet[e,f], Emmanuel Guiderdoni[e,f], Shaofei Wang[a,g], Yixiu Guo[a,g], Weiwei Zhang[a,g], Tianqi Wei[a,g], Yanan Huang[a,h], Dongli Hao[i], Jiajin Wang[a,g], Hervé Sentenac[b], Anne-Aliénor Véry[b,2,✉], Renfang Shen[a,2,✉], and Yanhua Su[a,j,2,✉]

[a] State Key Laboratory of Soil and Sustainable Agriculture, Institute of Soil Science, Chinese Academy of Sciences, Nanjing 211135, China

[b] Institute for Plant Sciences of Montpellier, University of Montpellier, Centre National de la Recherche Scientifique (Unité Mixte de Recherche 5004), Institut national de recherche pour l'agriculture, l'alimentation et l'environnement (Unité Mixte de Recherche 386), Institut Agro, 34060 Montpellier Cedex 2, France

[c] Shandong Institute of Sericulture, Yantai 264002, China

[d] National Key Laboratory of Crop Genetic Improvement, Huazhong Agricultural University, Wuhan 430070, China

[e] Centre de coopération internationale en recherche agronomique pour le développement (CIRAD), Unité Mixte de Recherche Amélioration Génétique et Adaptation des Plantes Méditerranéennes et Tropicales Institut, Montpellier F-34398, France

[f] University of Montpellier, Centre de coopération internationale en recherche agronomique pour le développement (CIRAD), Institut national de recherche pour l'agriculture, l'alimentation et l'environnement (INRAE), Institut Agro, Montpellier F-34398, France

[g] University of Chinese Academy of Sciences, Beijing 100049, China

[h] College of Ecology and Environment, Nanjing Forestry University, Nanjing 210037, China

[i] Jiangsu Key Laboratory for the Research and Utilization of Plant Resources, Institute of Botany, Jiangsu Province and Chinese Academy of Sciences (Nanjing Botanical Garden Mem. Sun Yat-Sen), Nanjing 210014, China

[j] University of Chinese Academy of Sciences, Nanjing 211135, China

[1]Present address: University of Glasgow, Laboratory of Plant Physiology and Biophysics and the Plant Science Group, School of Molecular Biosciences, Bower Building, Glasgow G12 8QQ, UK

[2]To whom correspondence may be addressed. Email: anne-alienor.very@cnrs.fr, rfshen@issas.ac.cn or yhsu@issas.ac.cn.

This PDF file includes:

Supplementary text
Figures S1 to S13
Supplementary movies 1 and 2
Tables S1 to S2
SI References

Materials and Methods

Molecular cloning and generation of plant materials

The coding sequences (CDS) of *OsKAT1* (LOC_Os01g55200), *OsAKT1* (LOC_Os0g45990), *OsCBL1* (LOC_Os10g41510), *OsCIPK23* (LOC_Os07g05620), and *AtKAT1* (At5g46240) were obtained by high-fidelity PCR amplification from cDNA templates of rice (*japonica* Nipponbare) and *Arabidopsis* (Columbia ecotype), respectively, and verified by sequencing. According to the sequence alignment illustrated in *SI Appendix,* Fig. S3*A,* the distal C-terminal sequences of AtKAT1 downstream of the putative cNBD domain (C1) was deleted by PCR amplification to generate a shortened version, designated AtKAT1△C1 (structural diagrams of all chimeras are provided in *SI Appendix,* Fig. S3*A*). To engineer chimeric channels with extended C-terminal regions resembling those of "classical" Shaker channels, the distal C-terminal segments from AtKAT1 (C1, amino acids 501-677) and OsAKT1 (C2, amino acids 541-935) were amplified by PCR and fused to OsKAT1. The resulting constructs were named OsKAT1-C1 and OsKAT1-C2, respectively. Additionally, the ankyrin (ANK) domain of OsAKT1 (amino acids 565-757) was amplified and inserted into the OsKAT1-C1 construct at the corresponding position to generate the chimera OsKAT1-C1-ANK. All CDS of the above-mentioned channels and reconstituted chimeras were cloned into the oocyte expression vector pCI. For *in vitro* cRNA synthesis, the coding sequences of OsCBL1 and OsCIPK23 were cloned into the pT7TS vector.

The promoter region of the *OsKAT1* gene (2264 bp, *pOsKAT1*) was amplified by PCR from genomic DNA of rice *cv*. Nipponbare, verified by sequencing, and subsequently was introduced into the plant expression vector pCAMBIA1301 upstream of the *GUS* reporter gene to replace the *CaMV-35S* promoter, resulting in the reporter construct *pOsKAT1:GUS*. Transgenic rice plants expressing GUS reporter gene were obtained using *Agrobacterium*-mediated transformation, following previously described method (1).

Furthermore, a plasmid construct was for expressing the C-terminal eGFP-tagged OsKAT1 protein under the control of its native promoter *pOsKAT1*, resulting in the fusion reporter construct *pOsKAT1:OsKAT1:eGFP*. To create OsKAT1 knockout mutants (*oskat1-1* and *oskat1-2*), targeted gene editing was performed using the CRISPR-Cas9 system. Briefly, a gene-specific spacer targeting to the CDS of OsKAT1 was selected from rice gene-specific spacer library (2). The sgRNA expression cassette was assembled *in vitro* by PCR amplification of the ligated cassette and subsequently introduced into the final expression vector pBWA (VH-Cas9ir)-*OsKAT1* using the Golden Gate technology (3,4). For the overexpression construct, the CDS of *OsKAT1* was inserted into the pUN1301 vector, a modified version of pCAMBIA1301 in which the CaMV 35S promoter was replaced by the ubiquitin promoter, as previously described by Wang et al. (5), yielding the expression plasmid pUN1301-OsKAT1. Rice transformations including for the *pOsKAT1:OsKAT1:eGFP* construct, were conducted by Wuhan Biorun Bio-Tech Co., Ltd (China). The plasmids were introduced into *Agrobacterium tumefaciens* strain EHA105 and subsequently

transformed into *calli* derived from mature seeds. For mutant and OsKAT1:eGFP lines, the recipient was *Oryza sativa* ssp. *Japonica* cv. Nipponbare (NB), while for overexpression (OE) line, Zhonghua11(ZH11) was used. Transgenic seedlings were regenerated through tissue culture (6). Nucleotide deletions in two independent CRISPR-Cas9 mutant lines were confirmed by sequencing (*SI Appendix,* Fig. 2*A*). The expression level of *OsKAT1* in *OE* lines (*SI Appendix,* Fig. 2*B*) was quantified by qRT-PCR using *OsActin* as the internal reference (primers listed in *SI Appendix,* Table S1). Homozygous lines were screened and selected for subsequent hydroponic and field experiments.

For functional expression in *Arabidopsis*, the coding sequences of *OsKAT1* and *OsKAT1-C1* were individually cloned into the plant expression vector pCAMBIA1301 under the control of the CaMV-35S promoter. Two parallel transformations were carried out in wildtype *Arabidopsis* (Columbia ecotype, Col-0) and the other in the *skor* T-DNA knockout (SALK_132944) background, using the floral dip method (7). Transgenic plants were screened on solid medium (0.8% agar) containing half-strength MS salts supplemented with 50 mg L^{-1} hygromycin, and positive transformants were confirmed by PCR. The expression levels of the transgenes *OsKAT1* and *OsKAT1-C1* in *Arabidopsis* roots were quantified by qRT-PCR and normalized to the expression of *AtActin*. All primers used in this study were listed in *SI Appendix, Table S1*.

The molecular cloning procedures in ths study utilized PCR reagents, high-fidelity polymerase, DNA ligases and related enzymes obtained from Vazyme Biotech Co., Ltd (Nanjing, China). Restriction enzymes (*Xho* I, *Not* I, *Mlu* I, *Bgl* II, *Spe* I, *Xma* I, *Sac* I, *Kpn* I, *Bam* HI, *etc*.) were purchased from New England Biolabs (USA). cDNA synthesis was performed using reverse transcriptase supplied by Invitrogen (USA).

Hydroponic experiments with rice lines

For hydroponic culture of rice plants, seeds of various genotypes were surface-sterilized and germinated in distilled water for 2 days. All seedlings were subsequently grown in an artificial climate chamber maintained at 27 / 25°C (day / night temperature) under a 16 h light / 8 h dark photoperiod. The light intensity was maintained at 400 μmol $m^{-2}s^{-1}$, and relative humidity was kept at 70%. The modified IRRI (International Rice Research Institute) nutrient solution containing 1 mM NH_4Cl,1 mM $NaNO_3$, 0.3 mM KH_2PO_4, 0.35 mM K_2SO_4, 1 mM $CaCl_2 \cdot 2\,H_2O$, 1 mM $MgSO_4 \cdot 7\,H_2O$, 0.5 mM Na_2SiO_3, 20 μM NaFeEDTA, 20 μM H_3BO_3, 9 μM $MnCl_2 \cdot 4\,H_2O$, 0.32 μM $CuSO_4 \cdot 5\,H_2O$, 0.77 μM $ZnSO_4 \cdot 7\,H_2O$ and 0.39 μM $Na_2MoO_4 \cdot 2\,H_2O$ (pH 5.8), with 1 mM K^+ was used for preculture. The solution was refreshed every 2 days. Five-day-old transgenic Nipponbare (NB) seedlings were used for OsKAT1 localization analysis; seven-day-old plants for semi-quantitative RT-PCR analysis; ten-day-old plants for qRT-PCR; and two- to three-week-old seedlings for physiological and salinity tolerance measurements. For K^+ treatment, KH_2PO_4 and K_2SO_4 in the nutrient solution were replaced equimolar NaH_2PO_4 and Na_2SO_4, respectively, and KCl was added to achieve the desired K^+ concentrations. An additional growth condition identical to Obata et al. (8, method cited from 9)

was employed to further verify OsKAT1 expression in rice.

For the collection of xylem and phloem sap, fourteen-day-old rice seedlings were first deprived of K^+ for 3 days, then re-supplemented with 20 mM K^+ for 3 h prior to sampling. Shoots were excised 3 cm above the root-shoot junction. Xylem sap was collected from the cut stumps using absorbent cotton over a 3-hour period. Phloem exudates were obtained by immersing the base of excised shoots in 5 mL of 20 mM EDTA for 3 h, following previously described methods (1, 7, 10, 11).

Photosynthetic parameters were measured using a LI-6400 portable photosynthesis system (Li-Cor, USA) according to the manufacturer's instructions. Fourteen-day-old seedlings grown in 1 mM K^+ nutrient solution were exposed to20 mM K^+ for 2 h before the measurement. The middle section of the second last leaf was used to determine stomatal conductance, photosynthetic rate and transpiration rate between 9:30 and 11:30 a.m. During the measurements, a saturating photosynthetic photon flux density (PPFD) of 1500 μmol $m^{-2}s^{-1}$ was supplied using an integrated LED light source, with CO_2 concentration set at 400 μmol mol^{-1} and flow rate at 500 μmol s^{-1}.

For ion content and stress assays, ten-day-old seedlings were grown for 7 additional days in nutrient solution containing 10 mM K^+ to assess K^+ accumulation and growth, or for fourteen days under 100 Mm NaCl to evaluate the role of OsKAT1 in salt stress tolerance. Biomass and ion content (K^+ and Na^+) were measured upon sample harvest.

Histochemical assays of GUS Activity

For histochemical localization of GUS activity, one-week-old homozygous *pOsKAT1:GUS* plants, screened on hygromycin resistance in the progeny, were grown in Yoshida hydroponic medium as described previously (12). Samples were immerged in GUS staining solution (50 mM phosphate buffer, pH 7.2, 5 mM potassium ferricyanide, 2.5 mM potassium ferrocyanide, 0.05% [v/v] Triton X-100, and 1 mM X-Gluc) in a 24-well plate. Infiltration was facilitated by applying vacuum for 30 min, followed by overnight incubation at 37°C. After staining, tissues were fixed using a two-step protocol: prefixation in 50 mM phosphate buffer (pH 7.0) containing 1.5% formaldehyde and 0.05% Triton X-100, followed by fixation in 75 mM phosphate buffer with 2% paraformaldehyde and 0.5% glutaraldehyde. Fixed samples were embedded in Technovit 7100 resin (Kulzer), and thin sections (8 μm thickness) were prepared using a RM 2165 microtome (Leica).

GFP fluorescence imaging and localization

Five-day-old rice seedlings expressing the *pOsKAT1:OsKAT1:eGFP* fusion construct were used to localize the eGFP-tagged OsKAT1 protein. Initially, whole seedlings were imaged under a low-light cooled charge-coupled device imaging system (Vilber, FUSION FX7 IR SPECTRA) to visualize the overall distribution of the fusion protein. Then root cross sections were prepared following the protocol described by Yamaji et al. (13). Briefly, fresh rice root samples were fixed in 4% (w/v) paraformaldehyde and 60 mM sucrose buffered with 50 mM cacodylic acid (pH 7.4) for 2 h at room temperature, with occasional degassing, followed by three washes with a solution containing 60 mM sucrose and 50 mM cacodylic acid (pH 7.4). Fixed tissues were embedded in 5% agar and

sectioned into 100-μm slices using a Microslicer (ZERO 1N, Dosaka). The sections were mounted on microscope slides, and GFP fluorescence was observed under a confocal microscope (LSM710, Zeiss, Germany). GFP signal was excited at 488 nm, and cell wall autofluorescence was detected at an excitation wavelength of 405 nm.

Immumo-localization analysis

Immuno-localization of OsKAT1:eGFP fusion protein was detected on 100- m-thick root cross-sections using an anti-GFP antibody. Sections were first treated with phosphate-buffered saline (PBS; 10 mM PBS, pH 7.4, 138 mM NaCl, 2.7 mM KCl) containing 0.1% (w/v) pectolyase Y-23 (KYOWA, CHEMICAL) at 25°C for 2 h, followed by incubation in PBS with 0.3% (v/v) Triton X-100 at 25℃ for 2 h. After each step. Sections were washed three times with 1×PBS. Subsequent immuno-histochemical staining was carried out according to manufacturer's instructions (Abcam ab64261). Briefly, endogenous peroxidase activity was quenched by applying 100 μL of hydrogen peroxide block to cover the sections and incubating for 10 minutes at 25°C. After three washes with PBS, nonspecific binding sites were blocked with 5% BSA in PBS. Sections were then incubated overnight at 25°Cwith a primary antibody solution (Invitrogen, rabbit anti-GFP antibodies, 1:1000 dilution in PBS), while control slides were incubated without primary antibody. After four washes with PBS, 100 μL of biotinylated goat anti-polyvalent secondary antibody was applied and incubated for 10 minutes at room temperature. After another four washes, streptavidin peroxidase was added and incubated for 10 minutes. Sections were thoroughly rinsed, and color development was initiated using diluted (1:50) DAB chromogen until desired staining intensity was achieved. After a final wash, counterstaining was performed with 100 μL of haematoxylin for 3 minutes. The stained sections were washed again, mounted in 40% glycerol and visualized under an optical microscope.

Plant experiments using *Arabidopsis* transformants

Seeds of *Arabidopsis* transgenic lines expressing OsKAT1 or the elongated chimera OsKAT1-C1 in either the Col-0 or *skor* mutant background were germinated and grown vertically on half-strength MS agar plates supplemented with 50 mg/L hygromycin. Plants were maintained for 5 days in an illuminated incubator (MMM, CLIMACELL, Germany) at 23°C under 16 h / 8 h light / dark cycle, with a light intensity of 100 μmol $m^{-2}s^{-1}$ and 70% relative humidity. Uniform seedlings from selected independent lines were subjected to grafting to confine transgene expression only to the roots (14). Heathy seedlings were transversely cut at the hypocotyl with a diamond dissection knife on cellulose nitrate filters (Millipore) under sterile conditions. Scions were carefully aligned onto rootstocks as previously described (14). Grafted plants were cultured for an additional 7 days on the plates. Successful grafts without adventitious roots were transplanted into rinsed soil matrix (7) and grown for 4 more weeks under the same environmental conditions. Plants were watered every 3 days with equal volumes of nutrient solution containing 1 mM K^+. Xylem sap was collected from

Arabidopsis grafts following Han's method (15). Shoots were excised 2 cm above the root-shoot junction, and the cut surface was cleaned with filter paper. Xylem exudate was harvested from excised rootstocks using glass capillaries within 2 h. Each biological replicate consisted of sap pooled from two plants, yielding approximately 200 µL per replicate. A total of six replicates were collected per genotype. The xylem sap samples were diluted in deionized water for K^+ concentration measurement. In parallel, K^+ content in fresh shoot and rinsed root tissues were determined after homogenization and extraction with 1 N HCl (shoots) or distilled H_2O (roots).

RNA extraction and qRT-PCR

Total RNA was extracted from rice and *Arabidopsis* plants using TRIzol reagent (Invitrogen, USA), followed by genomic DNA removal with a 4×gDNA wiper Mix as instructed (Vazyme, Nanjing, China). First-strand cDNA was synthesized using RevertAid First Strand cDNA Synthesis Kit (Invitrogen, USA) according to the manufacturer's instructions. PCR amplification was carried out with gene-specific primers, and the products were separated by electrophoresis on a 1.5% agarose gel for visualization. Quantitative real-time PCR (qRT-PCR) was performed using the ChamQ Universal SYBR qPCR Master Mix (Vazyme, Nanjing, China) on a QuantStudio 3 Real-Time PCR System (Applied Biosystems, Thermo Fisher Scientific). Gene expression levels were quantified using the $2^{-\Delta\Delta CT}$ method (16), with *OsActin* and *AtActin* serving as internal controls for rice and *Arabidopsis*, respectively.

Electrophysiological recordings in oocytes

Healthy oocytes were collected from mature *Xenopus laevis* frogs and prepared as previously described (17,18). The cRNAs of *OsCBL1* and *OsCIPK23* were synthesized *in vitro* using the T7 mMESSAGE mMACHINE Kit (AM1344, Thermo Fisher Scientific). Oocytes were micro-injected (Nanoliter 2000, WPI, Sarasota, FL, USA) with 60 ng plasmid DNA (18,19) carrying sequences encoding wild-type OsKAT1, AtKAT1, or their chimeric channels. For co-expression experiments with the OsCBL1/OsCIPK23 complex, 60 ng of channel plasmid DNA was co-injected with 30 ng each of *OsCBL1* and *OsCIPK23* cRNAs. Control oocytes were injected with an equivalent volume of H_2O or kinase cRNA alone. After injection, oocytes were incubated at 19°C for 48 h in ND96 solution (mM: 96 NaCl, 2 KCl, 1 $MgCl_2$, 1.8 $CaCl_2$, 5 HEPES, pH 7.4, supplemented with 0.05 mg/mL gentamycin). Two-electrode voltage-clamp recordings were performed using an Axoclamp 900A amplifier (Molecular Devices, San Jose, CA, USA). The standard bath solution contained 100 mM NaCl, 1.8 mM $CaCl_2$, 1 mM $MgCl_2$, 5 mM HEPES (pH 7.4). For K^+ current measurements, KCl was added at specified concentrations by equimolar substitution of NaCl. Ion selectivity was assessed using bath solutions containing 100 mM of NaCl, LiCl, RbCl or KCl. The pH regulation of currents was evaluated under 50 mM K^+ with the recording solutions buffered to either pH7.4 (5 mM HEPES/NaOH) or pH 5.4 (5 mM MES/NaOH). Channel blockade was tested in the presence of 5 mM $BaCl_2$ under 50 mM K^+ at pH 7.4. Voltage steps from -160 mV to +20 mV in +10 mV increments were applied from a holding potential of -40 mV.

Patch-Clamp recordings with root stele protoplasts of rice

Mature roots were excised from 7-day-old seedlings of wildtype (cv. Nipponbare) and *oskat1* mutant rice for the isolation of stele cell protoplasts. Briefly, after removing the epidermis and part of the cortical tissues, the remaining root material was finely chopped. Isolation of stele cell protoplasts and subsequent patch-clamp recordings were carried out based on established methods previously described for barley (20) and maize (21). Whole-cell K^+ currents were recorded using an Axopatch 200B amplifier (Molecular Devices). The pipette solution contained (in mM): 120 K-gluconate, 1 $CaCl_2$, 2 $MgCl_2$, 2 Tris-ATP, 10 Tris, 10 EGTA, pH 7.2 (adjusted with MES), with D-sorbitol added to achieve an osmolarity of 550 $mOsm \cdot L^{-1}$. The bath solution consisted of (in mM): 10 KCl, 1 $CaCl_2$, 2 $MgCl_2$, 10 MES, pH 5.8, supplemented with D-sorbitol to adjust the osmolarity to 500 $mOsm \cdot L^{-1}$. Voltage protocols consisted of 1.5-second step pulses from -180 mV to +100 mV in 10 mV increments, with a 1.5-second holding period at -40 mV between steps.

Field experiments

Field experiments were carried out over two growing seasons 2017 and 2019) in a typical paddy field at the State Experimental Station of Agro-Ecosystem in Changshu, Chinese Academy of Sciences (31°15′15″ N, 120°57′43″ E). The region experiences a northern humid subtropical monsoon climate, with a mean annual temperature of 17°C and an average annual precipitation of 1200 mm. During the rice flowering and grain-filling stages (July to September), typical daily temperatures range from 31 to 36°C during the day and 24 to 27°C at night. The topsoil (0-20 cm) had an initial pH of 5.2 and relatively low levels of available nutrients, with nitrogen (N) at 7.3 ppm, phosphorous (P) at 7.0 ppm and potassium (K) at 70 ppm. Fertilizers were applied at rates of 200 kg N, 90 kg P_2O_5 and 240 kg K_2O per hectare during each growing season to ensure adequate nutrient supply. Homozygous T3 and T4 generation plants were arranged in randomized plots measuring 5 m × 4 m, with a planting density of 20 cm × 25 cm. K^+ and N contents in flag leaves or whole plants were measured during the grain-filling stage, and agronomic traits were evaluated at harvest, as previously described (22).

Determination of K, Na and N content

Plant and leaf samples were dried, ground into a fine powder, and digested with H_2SO_4-H_2O_2. The K^+ and Na^+ concentrations in the digests were quantified by flame spectrophotometry (Sherwood, M410). Calibration was performed using standard curves generated from serial dilutions of solutions with known concentrations of K^+ or Na^+. K and Na contents per plant were derived from these measurements.. The K accumulation per plant (in mg or g per plant) was calculated as follows: plant K content × dry weight per plant. N content was determined by an elemental analyzer (SmartChem 200, Westco).

Data analysis and Statistics

Electrophysiological data were analyzed using Clampfit 10.3 (Molecular Devices). Graphs were

generated with SigmaPlot 12.5 and GraphPad Prism 8.0.2. Data are presented as means ± standard error (SE). Statistical significance was assessed with SPSS 11.0, employing two-tailed *Student's* t-tests or one-way analysis of variance (ANOVA) followed by Duncan's multiple comparison test.

Fig. S1

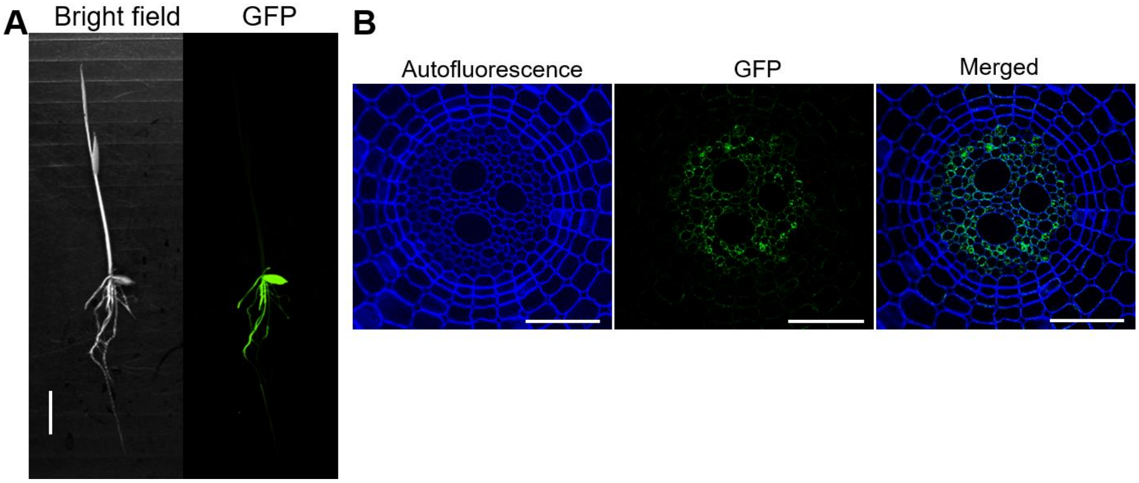

Fig. S1. Fluorescence analysis of OsKAT1 expression in transgenic rice seedlings. Five-day old transgenic rice seedlings (*cv.* Nipponbare) expressing the OsKAT1:eGFP fusion gene under the control of the native *OsKAT1* promoter were subjected to fluorescence imaging. (*A*) Representative seedling image acquired with a low-light cooled charge-coupled device imaging system (Vilber, FUSION FX7 IR SPECTRA). GFP fluorescence was excited at 488 nm. Scale bar: 1 cm. (*B*) Confocal micrographs of cross-sections from the root mature region (LSM710, Zeiss). Left, cell wall autofluorescence under 405 nm excitation; Middle, GFP fluorescence signal from OsKAT1:eGFP fusion protein (excitation 488 nm); Right, merged image of GFP fluorescence signals. Scale bar: 50 µm.

Fig. S2

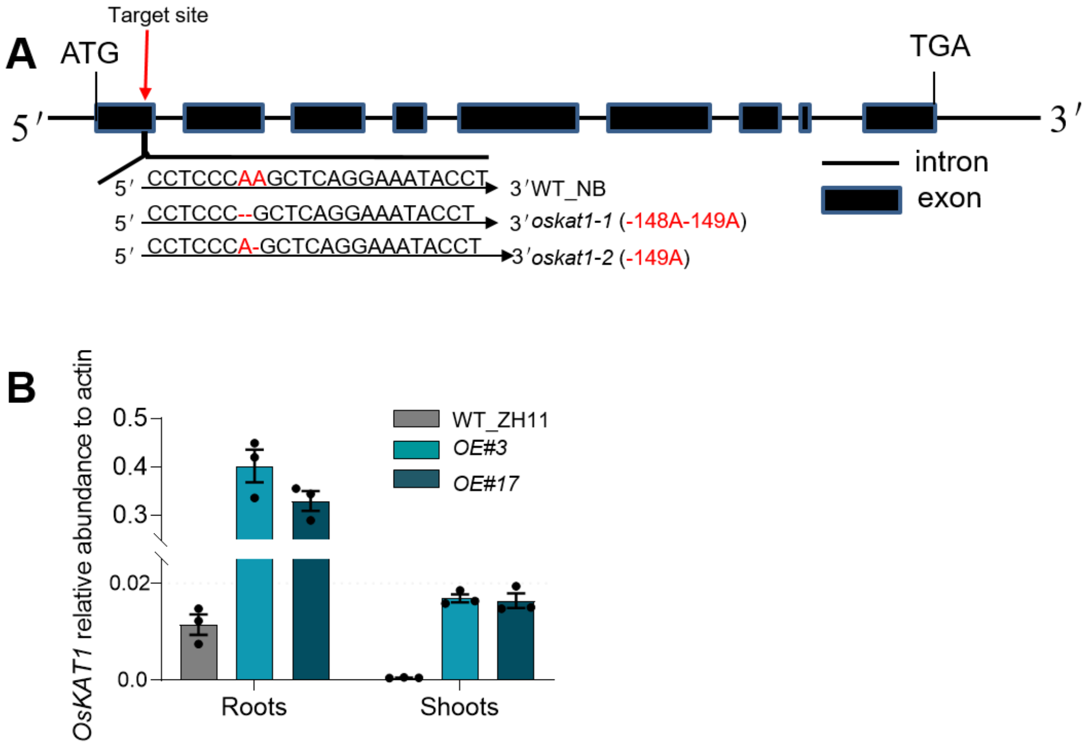

Fig. S2. Description to the rice genetic lines used in this work.

(*A*) Schematic illustration of the OsKAT1 gene structure in rice genome. CRISPR-Cas9 was used to introduce nucleotide deletions within the first exon, Yielding two independent knockout (KO) mutant lines in the *japonica cv.* Nipponbare background: *oskat1-1* (2-nt deletion at positions148 and149) and *oskat1-2* (1-nt deletion at position149). These mutations cause early termination of OsKAT1 protein translation at residues 58 and 69, respectively, both situated within the S1 transmembrane domain of OsKAT1. (*B*) Relative expression levels of OsKAT1 in two overexpression lines, *OE#3* and *OE#17,* generated in the Zhonghua-11 (ZH11) background. Homozygous KO and *OE* lines were propagated, screened and used for subsequent physiological analysis.

Fig. S3

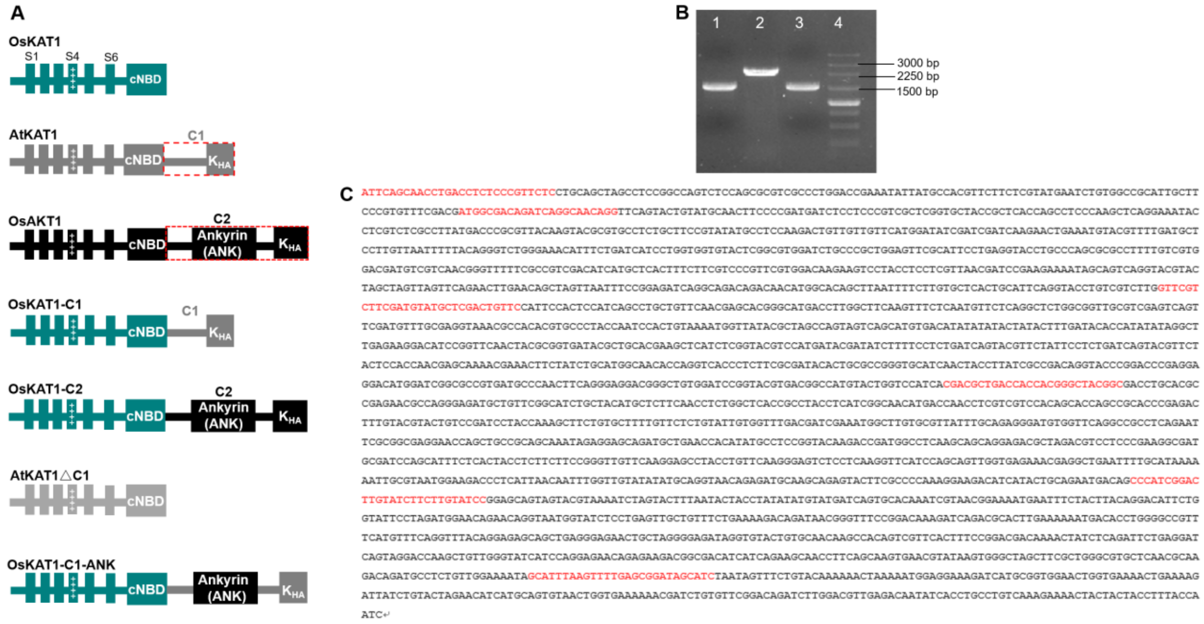

Fig. S3. Predicted structures of Shaker channels and validation of the full-length OsKAT1 coding sequence.

(*A*) Schematic diagrams of the predicted protein structures of OsKAT1, AtKAT1, OsAKT1 and recombinant chimeric channels. All Shaker channels display a hydrophobic core composed of six transmembrane segments (S1 - S6). The S4 segment contains positively charged residues and serves as the voltage sensor. A putative cyclic nucleotide-binding domain (cNBD) is present downstream of the transmembrane region. In both AtKAT1 and OsAKT1, a C-terminal region containing the so-called K_{HA} domain (enriched in hydrophobic and acidic residues and implicated in membrane clustering, 23), is present downstream of the cNBD. OsAKT1 additionally contains an ankyrin (ANK) domain between the cNBD and K_{HA} domains, which facilitates interactions with regulatory proteins. The C-terminal regions downstream of the cNBD in AtKAT1 and OsAKT1, absent in OsKAT1, are designated C1 and C2, respectively, in this study. (*B*) Validation of the full-length *OsKAT1* coding sequence. High-fidelity PCR amplification using primers flanking the star and stop codons produced fragments of 1509 bp from cDNA (lane 1) and 2665 bp from genomic DNA (lane 2), consistent with the annotated gene size and sequence. A subsequent PCR using a 5'-ATG forward primer and a 3'-oligo(dT)$_{30}$ reverse primer with cDNA template yielded a ~1.5 kb product (lane 3) containing the complete open reading frame encoding 502 amino acids, as confirmed by sequencing. (*C*) Primer walking sequencing of genomic DNA further validated the OsKAT1 gene structure. Overlapping primers (highlighted in red) were used to sequence a 2665 bp genomic fragment that matched the database annotation. These results confirm that, although C-terminally shorter than typical Shaker channels, OsKAT1 is a full-length Shaker K^+ channel encoded by a complete open reading frame.

Fig. S4

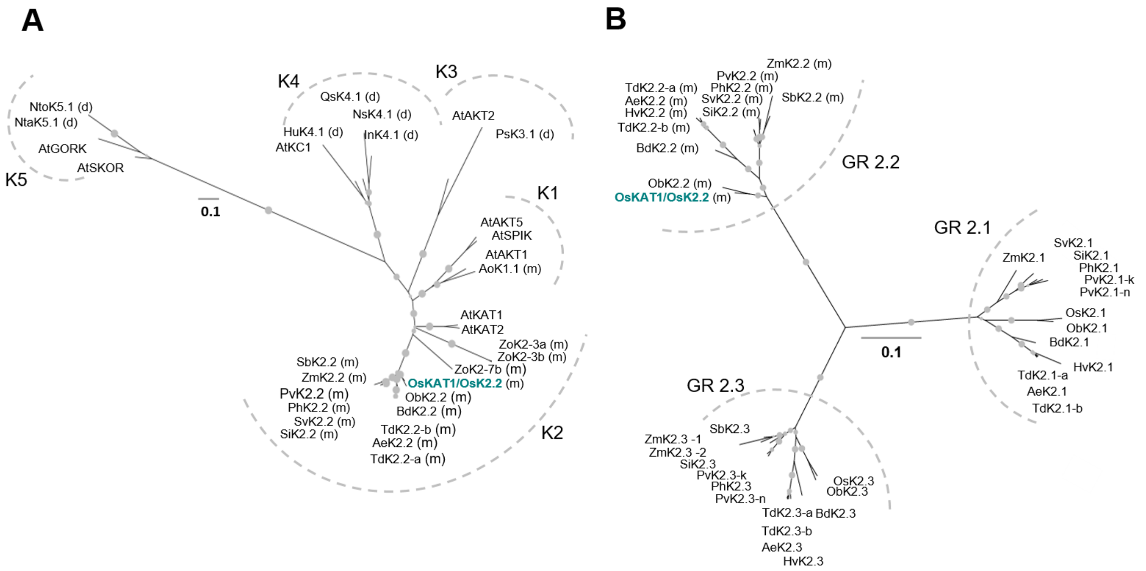

Fig. S4. OsKAT1 represents a distinct clade of short Shaker K⁺ channels prevalent in *Poales*. (*A*) Phylogenetic relationships between the nine Shaker channel proteins from *Arabidopsis* and native "short" Shaker polypeptides encoded by genes whose full-length open reading frame gives rise to a polypeptide displaying a C-terminal region devoid of K_{HA} and ANK domains. Short Shaker sequences were identified *in silico* from 1920 *Viridae* Shaker family members using BLASTP (see main text). "(d)" and "(m)" indicate dicot and monocot, respectively. Shaker family classification (K#1—K#5) follows the nomenclature proposed by Pilot et al. (24). Sequences were aligned with Muscle (v3.8.31) (25), and conserved blocks were selected with GBlocks (0.91b) (26) in Seaview (27). An unrooted maximum-likelihood phylogenetic tree was constructed using PhyML (version 3.3.3:3.3.20190909-1) (25) with 1000 bootstrap replicates. The phylogenetic tree was drawn with iTOL (https://itol.embl.de/upload.cgi); bootstrap values are indicated by grey circles scaled by support level. Scale bar: 10 changes per 100 amino acids. (*B*) Phylogenetic relationships of Shaker subfamily #2 channels from *Poales* species encoding a "short" Shaker sequence (excluding *Zingiber officinale*). The tree includes 11 plant species (including rice) identified in panel (*A*) and barley (see text). The unrooted phylogenetic tree, constructed as in (*A*), resolves 3 groups: GR2.1 to GR2.3. GR2.2 exclusively contains all and only short channels with the absence of a distal C-terminal region. Scale bar: 10 changes per 100 amino acids.

Species abbreviations:

Ae: *Aegilops tauschii*; Ao: *Asparagus officinalis*; At: *Arabidopsis thaliana*; Bd: *Brachypodiumdistachyon*; Hu: *Herraniaumbratica*; Hv: *Hordeum vulgare*; In: *Ipomoea nil*; Ns: *Nicotiana sylvestris*; Nta: *Nicotiana tabacum*; Nto: *Nicotiana tomentosiformis*; Ob: *Oryza brachyantha*; Os: *Oryza sativa*; Ph: *Panicum hallii*; Ps: *Papaver somniferum*; Pv: *Panicum virgatum*; Qs: *Quercus suber*; Sb: *Sorghum bicolor*; Si: *Setariaitalica*; Sv: *Setariaviridis*; Td: *Triticum*

dicoccoides; Zm: *Zea mays*; Zo: *Zingiber officinale*. Accession numbers are provided in *SI appendix*, *Table S2.* Note that OsKAT1 is also designated ‘OsK2.2’ in this phylogenetic classification.

Fig. S5

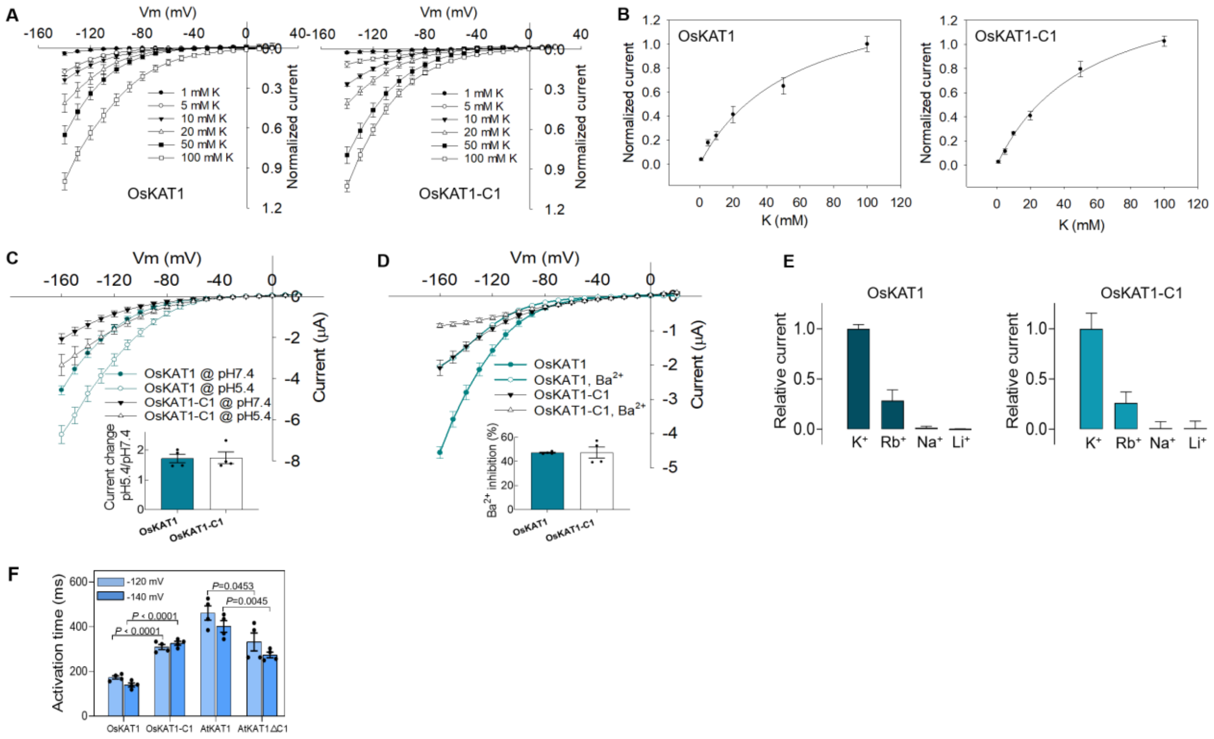

Fig. S5. Functional comparison of OsKAT1 and its C terminal extended variant OsKAT1-C1 in *Xenopus* oocytes.

(A) Current-voltage (I-V) relationships of OsKAT1 (left) and OsKAT1-C1 (right) under various K^+ concentrations. Currents were normalized to the average current amplitudes measured at -140mV in 100 mM K^+ for each channel type (n=6 for OsKAT1; n=3 for OsKAT1-C1). (*B*) Michealis-Menten kinetics of OsKAT1(left) and OsKAT1-C1 (right) at -140 mV. The deduced K_m values were of 56.6 mM for OsKAT1 and 54.3 mM for OsKAT1-C1, respectively. Normalized current amplitudes were derived from *A*. (*C*) pH sensitivity of OsKAT1 and OsKAT1-C1. Currents were recorded in 50mM K^+ at either pH 7.4 or 5.4. Inset: the pH effect was evaluated by ratios of $I_{pH5.4}$ / $I_{pH7.4}$ measured at -140 mV (n=4). (*D*) Ba^{2+} sensitivity of OsKAT1 and OsKAT1-C1. Currents were measured in 50 mM K^+, pH7.4 with or without 5 mM $BaCl_2$. Inset: inhibition rate at -140 mV (n=4). (*E*) Ionic selectivity of OsKAT1 and OsKAT1-C1. Macroscopic currents were measured at -140 mV in solutions containing 100 mM KCl, RbCl, NaCl or LiCl (pH 7.4) and expressed relative to that of 100 mM K^+ (n=4-6). Data are means ± SE. (*F*) Channel activation time constants at -120 and -140 mV. The time constants were obtained by fitting the current traces recorded at -120 and-140 mV to a single decaying exponential equation. Means ± SE (n=4). Statistical analysis: two-tailed *Student's* t-tests, *, P < 0.05; **, P < 0.01.

Fig. S6

A

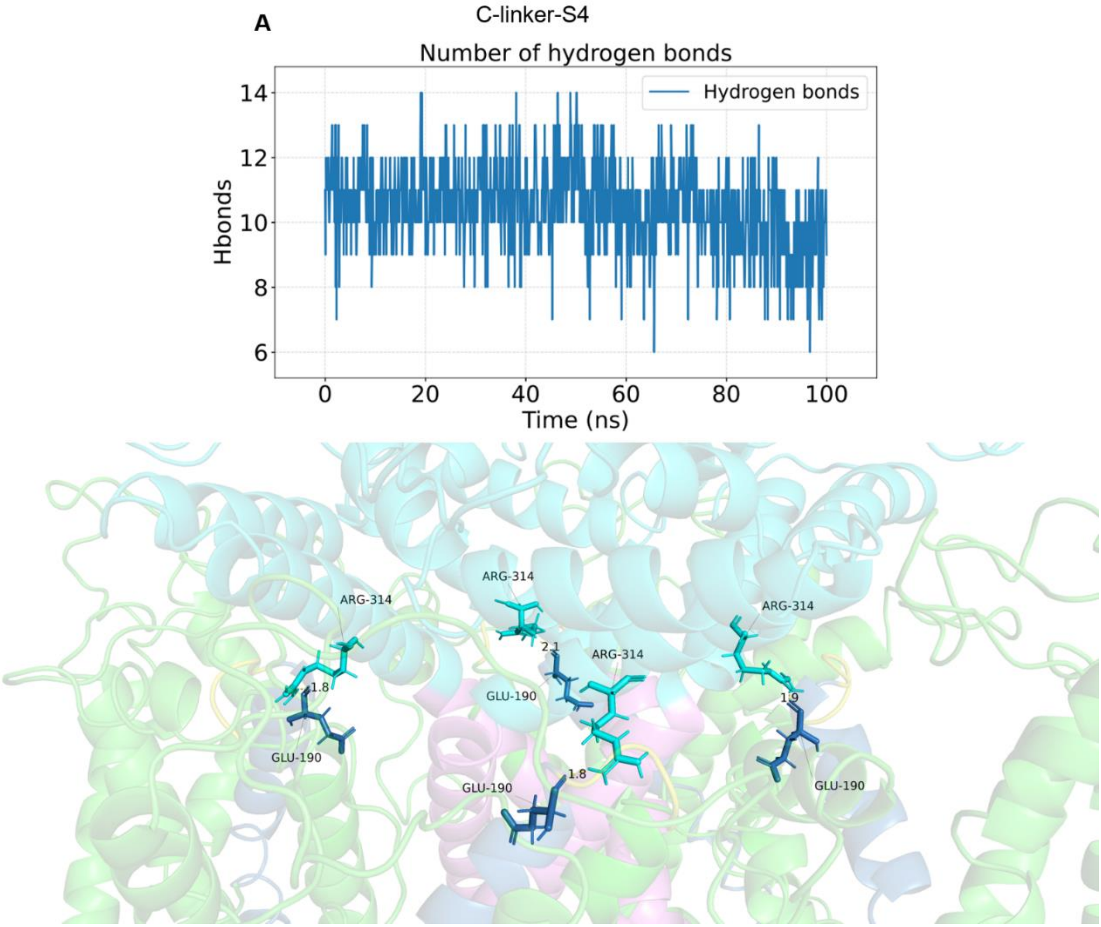
C-linker-S4
Number of hydrogen bonds
Hydrogen bonds
Hbonds
Time (ns)
14
12
10
8
6
0
20
40
60
80
100
ARG-314
ARG-314
ARG-314
ARG-314
GLU-190
GLU-190
GLU-190
GLU-190
1.8
2.1
1.9
1.8

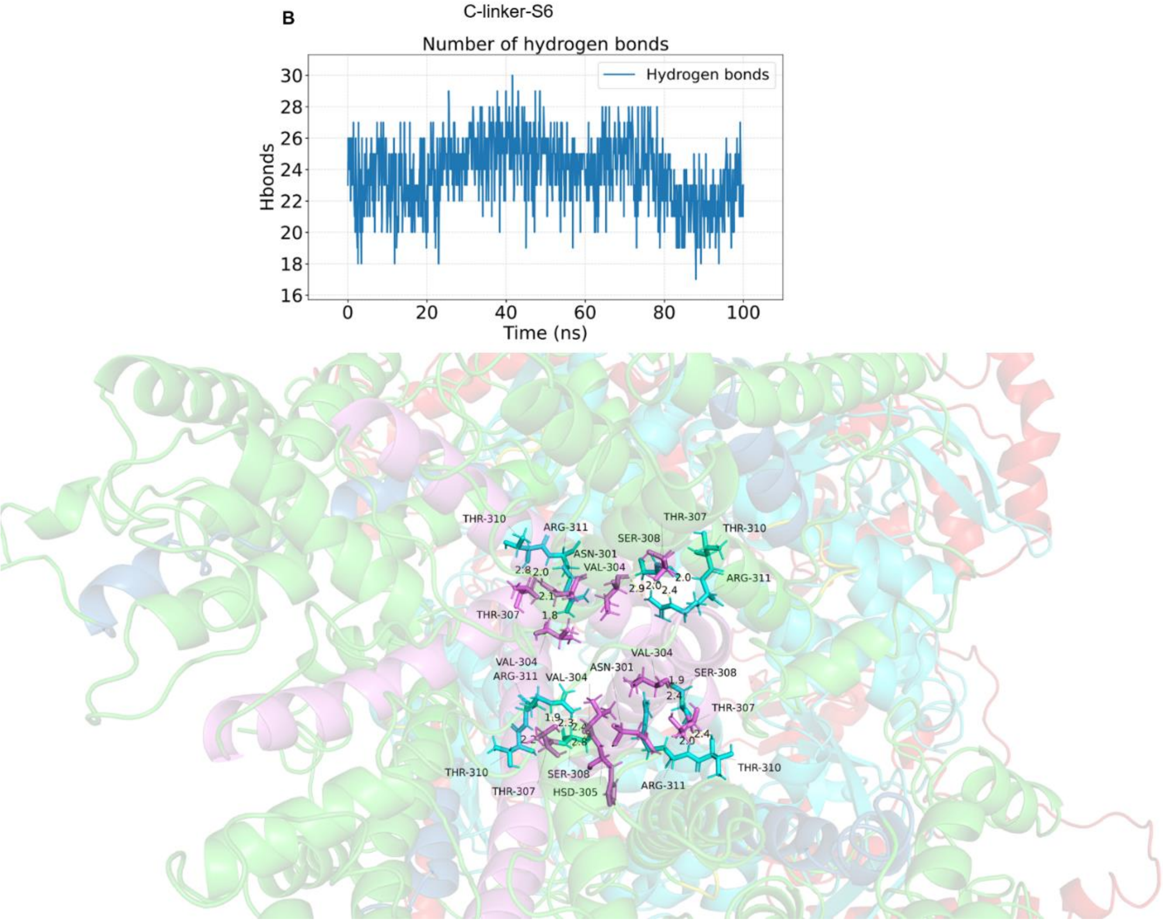

Fig. S6. Predicted hydrogen-bond interactions between the C-linker-S4 and C-linker-S6. Molecular dynamics simulations conducted over 100 ns suggest stable H-bond interactions between the C-linker motif with both S4 (A) and S6 (B) segments. In panels A and B, the number of hydrogen bonds is plotted over the simulation time course (upper), and annotated at the 100 ns time point (bottom).

Fig. S7

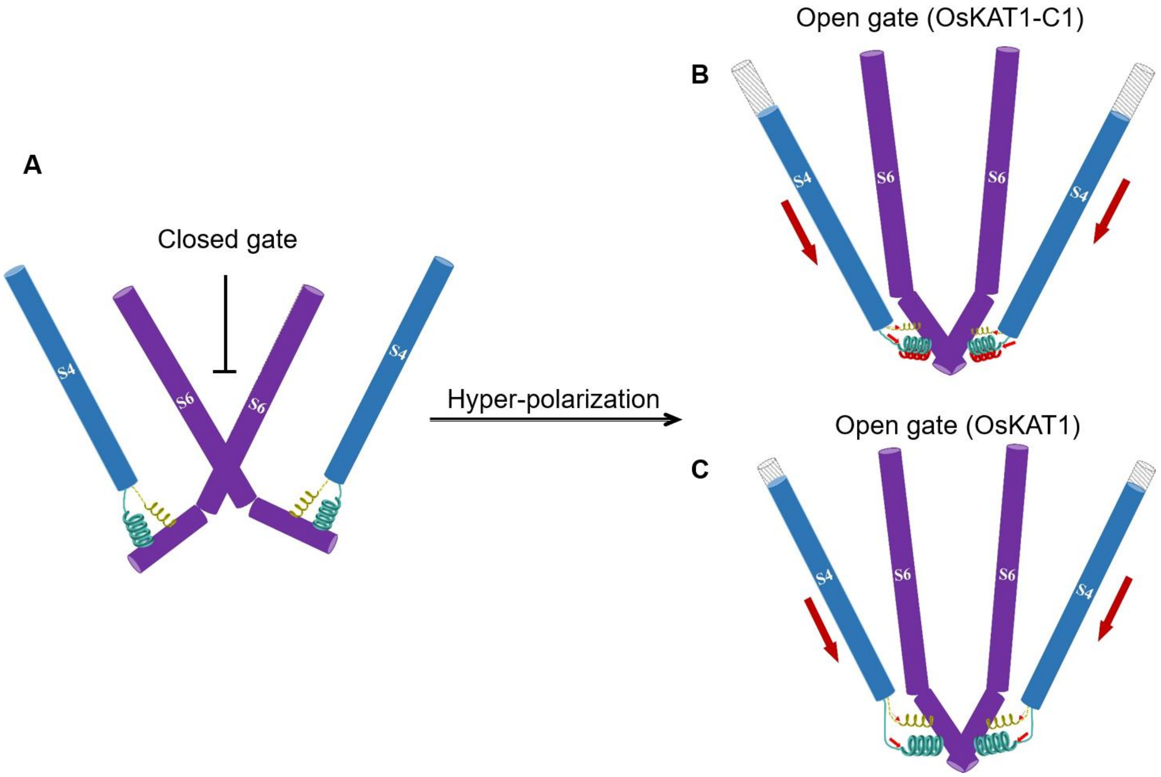

Fig. S7. Proposed models of channel activation.

Cartoon illustrations are adapted from the existing activation model of the AtKAT1 channel (28, 29). This model proposes that membrane hyperpolarization triggers an inward movement of the S4 helix, which through the force-transmission complexes, induces twisting of the S6 helix and subsequent opening of the channel gate. For clarity, the S4 and S6 helices from the four subunits are shown as colored cylinders in a plan view: S4 in sky blue and S6 in violet. (A) Under non-hyperpolarized conditions, the initial positions of the S4 and S6 helices maintain the channel gated in a closed state. (B and C) The S4 helix senses membrane hyperpolarization and moves inwardly. Because of no direct physical interaction between S4 and S6, the S4-S5 linker and, more importantly, the C-linker are predicted to play essential roles in transmitting force between these helices. (B) In the "full-length" channel OsKAT1-C1, the C1-term helix forms additional interactions with the C-linker, thereby impeding force transmission from S4 movement. As a result, a greater inward displacement of S4 is required to twist the S6 helix and open the gate, leading to channel open at more hyperpolarized membrane voltages. (C) In the short channel OsKAT1 with the absence of a C1 terminus, force transmission is simply mediated by the C-linker and the S4-S5 linker helices, resulting in reduced inward displacement of S4. Consequently, the channel opens at less hyperpolarized voltages. The C-linker, S4-S5 linker and the C1-term helix structures are colored in cyan, dark yellow and red, respectively.

Fig. S8

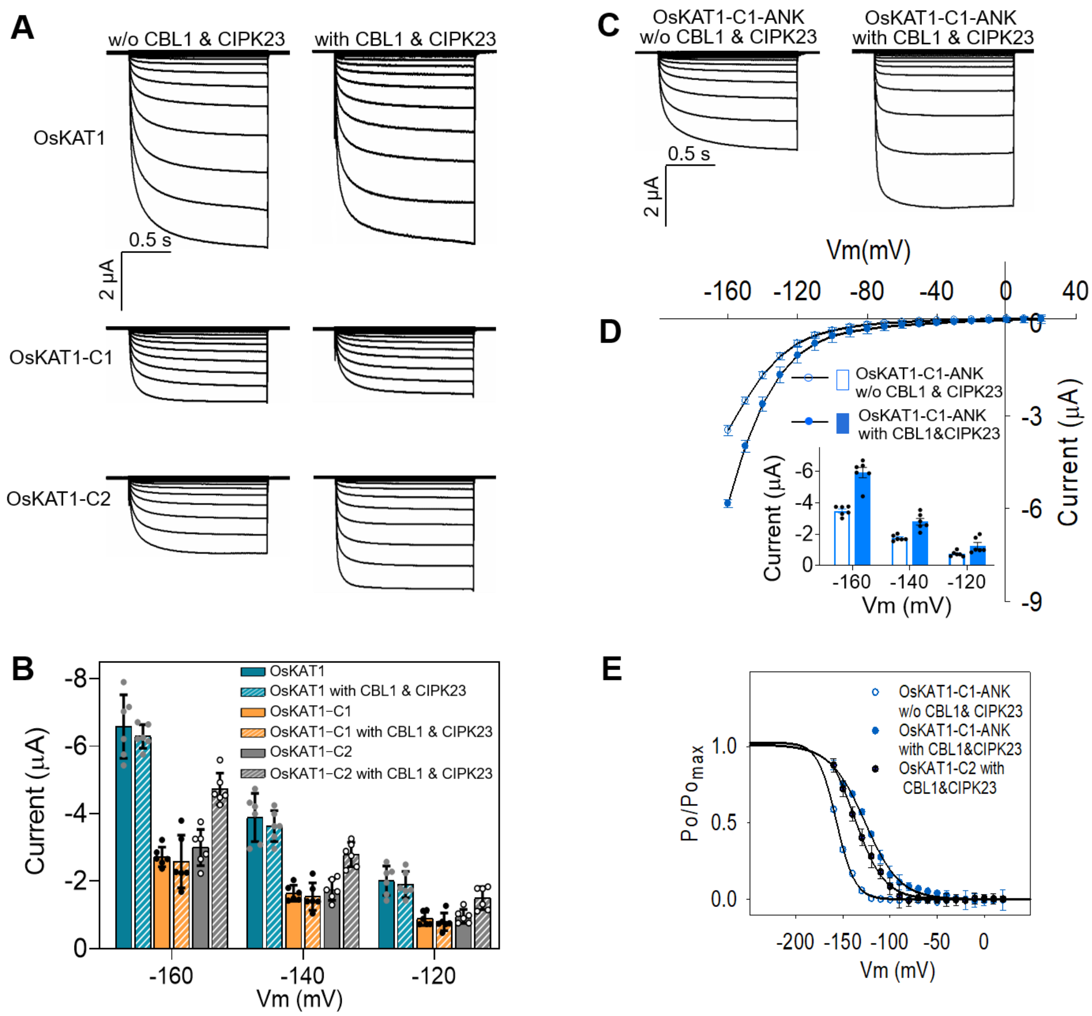

Fig. S8. Functional modulation of C-terminal extended OsKAT1 chimeras by the OsCBL1/OsCIPK23 complex.

OsKAT1 and derived chimeric constructs were expressed either alone or co-expressed with OsCBL1 and OsCIPK23 in oocytes. Electrophysiological recordings were conducted in 50 mM external K^+ (pH7.4). (*A*) Representative current traces from oocytes expressing OsKAT1, OsKAT1-C1 or OsKAT1-C2 alone (left) or co-expressed with OsCBL1 and OsCIPK23 (right). (*B*) Statistical summary of currents measured at -120, -140 and -160 mV. Data are means ±SE; n = 6. (*C-E*) The presence of the ANK domain in the OsKAT1-C1-ANK chimera confers sensitivity to the OsCBL1/OsCIPK23 complex. (*C*) Representative current traces from oocytes expressing OsKAT1-C1-ANK alone (left) or together with OsCBL1/OsCIPK23 (right). (*D*) Corresponding current-voltage relationships and statistical analysis (inset) of currents at -120, -140 and -160 mV. Means ± SE; n=6. (*E*) Gating properties in the absence and presence of the OsCBL1/OsCIPK23 complex. Voltage-dependency of relative channel open probability (Po/Po_{max}) was analyzed using a Boltzmann fitting. Means ± SE, n= 3, two-tailed *Student's* t-tests.

Fig. S9

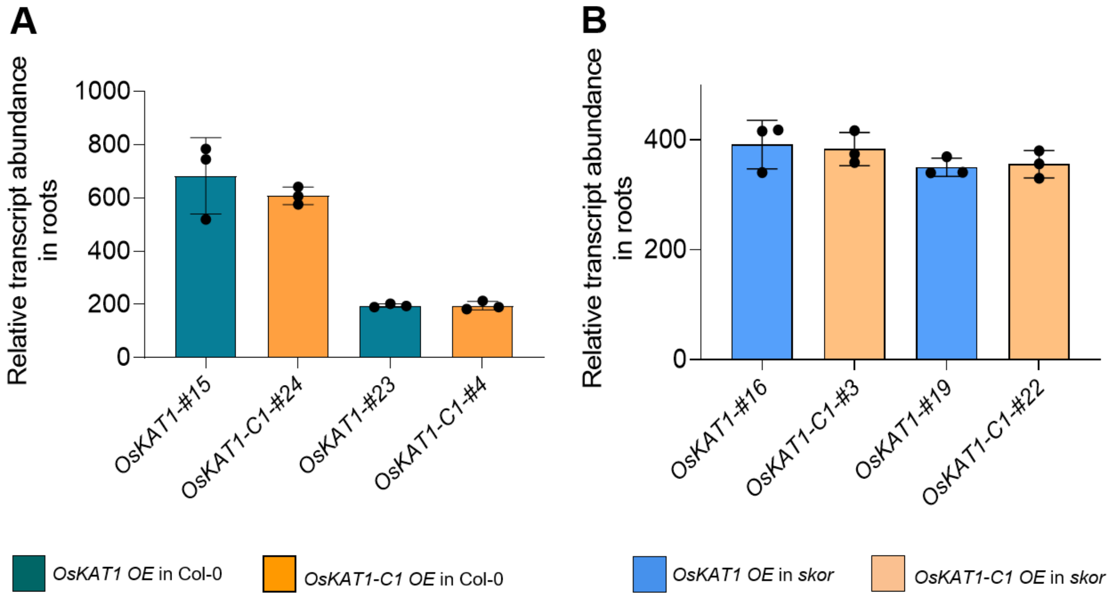

Fig. S9. Identification of transgenic *Arabidopsis* lines for phenotypic analyses. Transcript levels of transgenes in roots of 5-day-old *Arabidopsis* seedlings were quantified by qRT-PCR using *AtActin* as the reference gene. Seedlings were selected for grafting based on these expression results.

(*A*) In the Col-0 background (with the presence of the outward SKOR activity), two pairs of transgenic lines with comparable root expression levels of OsKAT1 and OsKAT1-C1 were identified: *OsKAT1-#15 vs OsKAT1-C1-#24* and *OsKAT1-#23 vs OsKAT1-C1-#4*. (*B*) In the *skor* mutant background (with the absence of the outward SKOR activity), two additional pairs showing similar transgene expression in roots were selected: *OsKAT1-#16 vs OsKAT1-C1-#3* and *OsKAT1-#19 vs OsKAT1-C1-#22.*

Fig. S10

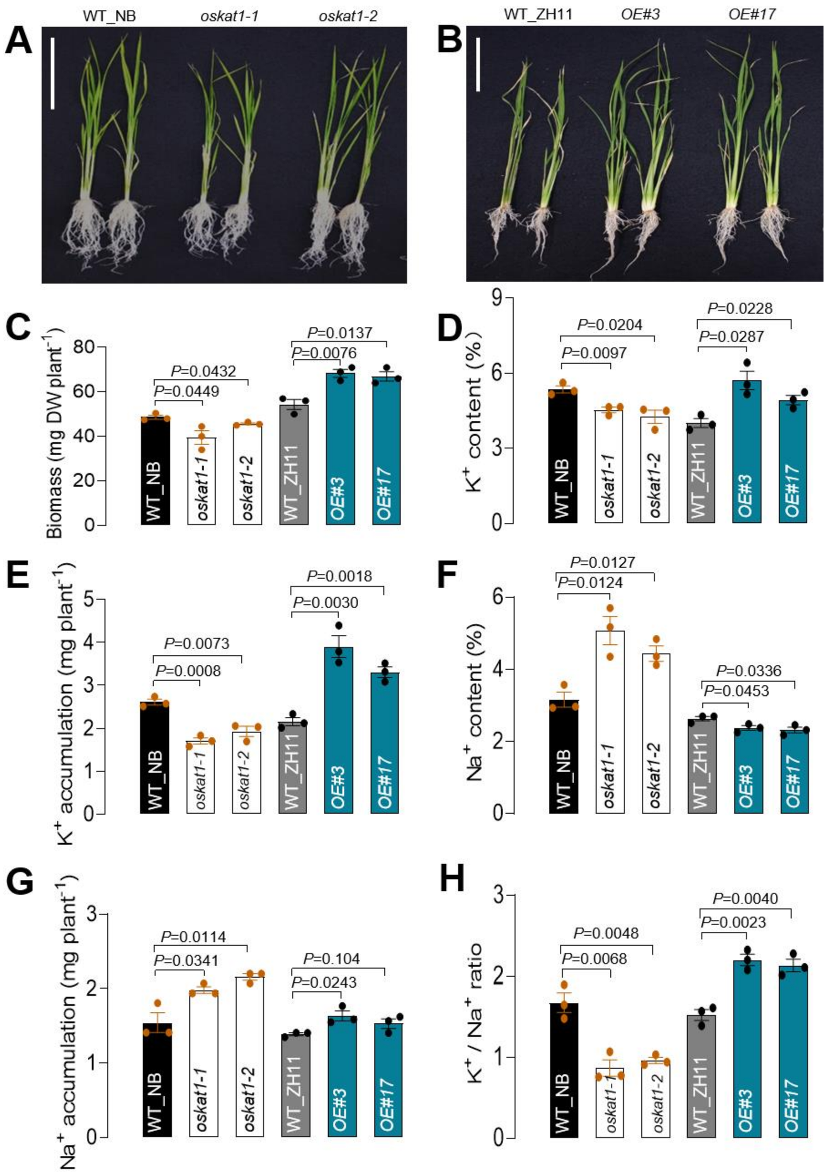

Fig. S10. OsKAT1 contributes to rice adaptation to saline conditions. Ten-day-old rice seedlings precultured in IRRI nutrient solution (1 mM K^+) were subjected to a salt stress treatment for 14 days in the presence of 100 mM NaCl. (*A* and *B*) Phenotypes of rice plants under NaCl stress. (A) shows *Nipponbare* KO mutants (*oskat1-1* and *oskat1-2*) and the wildtype control (WT_NB); (*B*) shows Zhonghua-11 overexpression lines (*OE#3* and *OE#17* lines) and their wildtype control (WT_ZH11). Scale bar: 10 cm. (*C*) Shoot biomass after 14 days of NaCl treatment. (*D*) Shoot K^+ content at the end of the treatment. (*E*) Total shoot K^+ accumulation (calculated as shoot biomass × shoot K^+ content). (*F*) Shoot Na^+ content. (*G*)Total shoot Na^+ accumulation. (*H*) Estimated ratio of shoot K^+ to Na^+ content. Means ± SE (n=3). Statistical analysis: two-tailed *Student's* t-tests, *, P < 0.05; **, P < 0.01.

Fig. S11

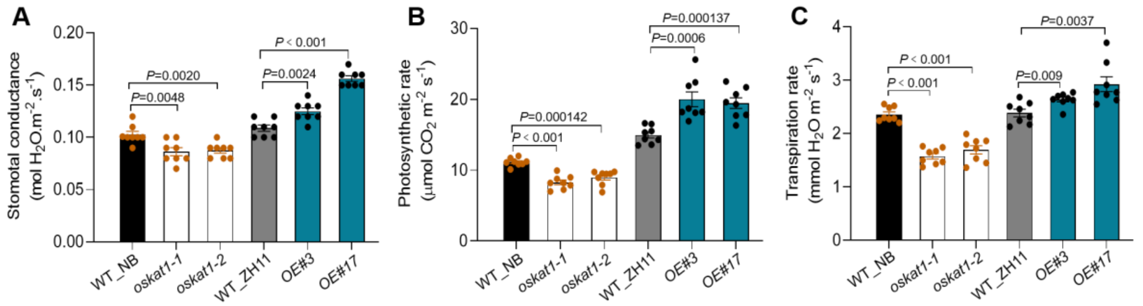

Fig. S11. Stomatal conductance, photosynthesis, and transpiration are influenced by OsKAT1 expression. Fourteen-day-old rice seedlings of *Nipponbare* wildtype (WT_NB) and *oskat1-1* and o*skat1-2* mutants, and Zhonghua11 wildtype (WT_ZH11), and overexpression lines *OE#3* and *OE#17*) were subjected to K^+ depletion for 3 days, followed by resupply with 20 mM K^+. Gas exchange parameters were measured using a Li-Cor LI-6400 portable photosynthesis system on the 2nd last leaves 2 h after K^+ resupply. (*A*) Stomatal conductance. (*B*) Net photosynthetic rate. (*C*) Transpiration rate. Data are means ± SE (n = 8). Statistical analysis: two-tailed *Student's* t-tests, *, P < 0.05; **, P < 0.01.

Fig. S12

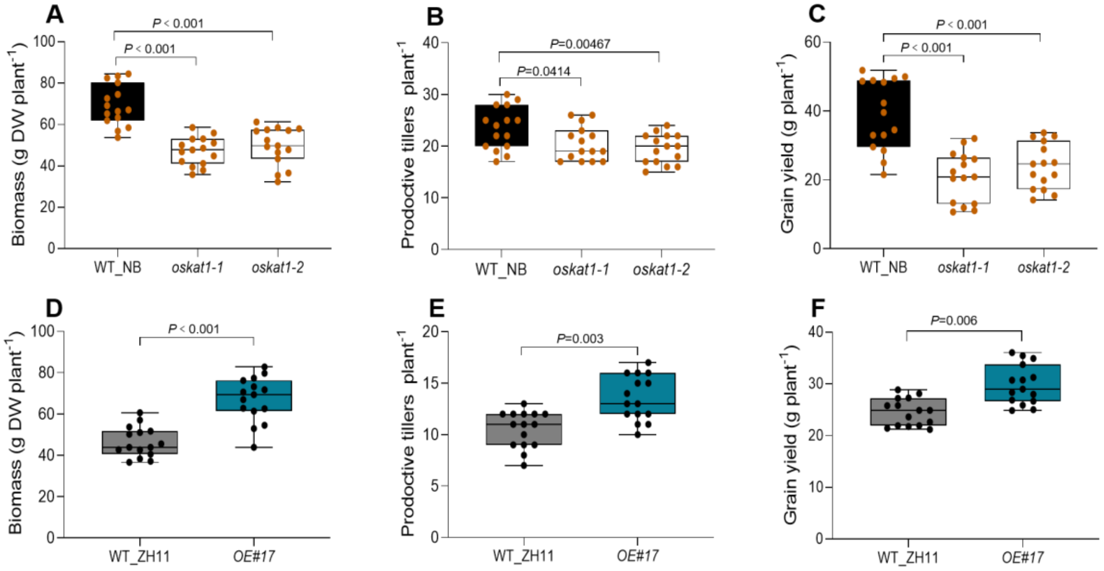

Fig. S12. Analysis of yield-related traits for the 2017 field experiments. Homozygous T3 generation plants were grown under field conditions, with fertilizer application rates of 200 kg N, 90 kg P_2O_5 and 240 kg K_2O per hectare. Agronomic traits were evaluated at harvest. (*A*-*C*) Plant dry biomass (*A*), number of seeding tillers per plant (*B*), and grain yield per plant (*C*) in *oskat1-1* and *oskat1-2* mutant lines. (*D*-*F*) Plant dry biomass (*D*), number of seeding tillers per plant (*E*), and grain yield per plant (*F*) in an OsKAT1-overexpression line. Means ± SE (n=15). Box plot shows maxima, first quartile, median, third quartile and minima. Statistical analysis: two-tailed *Student's* t-tests, *, P < 0.05; **, P < 0.01; ***, P < 0.001.

Fig. S13

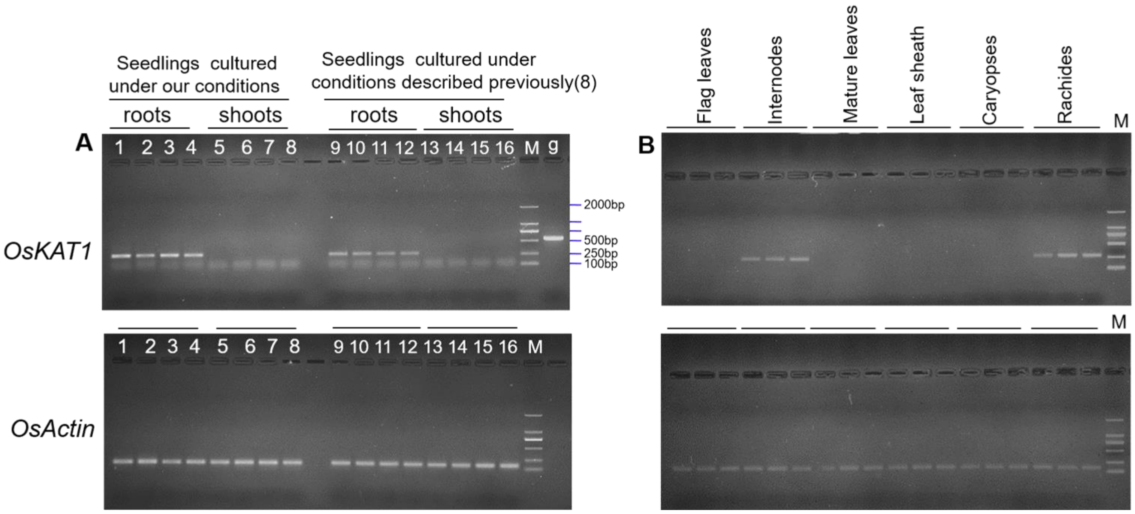

Fig. S13. Reconciliation of discrepant OsKAT1 expression pattern in rice. The root-stele predominant expression of OsKAT1 described in this study contradicts a previous claim that it is undetectable in young seedlings including roots (8). To address this discrepancy, we carried out additional semi-quantitative RT-PCR analyses using primers from this study with rice seedlings cultured under both experimental conditions. (*A*) Semi-quantitative RT-PCR analysis of OsKAT1 expression in 7-d-old rice seedlings (*cv.* Nipponbare). Plants were grown under the hydroponic conditions used in this study (lanes 1-8) or under previously reported conditions (8, as adapted from ref. 9; lanes 9-16). Four biological replicates of root and leaf tissues from each condition were analyzed; each sample consisted of tissue pooled from two individual plants. M: DL2000 DNA marker (TaKaRa); g: amplification from genomic DNA. Gene-specific primers (SI Appendix, Table S1) were designed to span three introns in the 3′ - region to avoid fault amplification from possible genomic DNA contamination. Expected product sizes were 275 bp for cDNA and 547 bp for gDNA templates, respectively. PCR conditions were: 95 °C for 5 min; 35 cycles of 95 °C for 30 s, 60 °C for 30 s, and 72 °C for 40 s; followed by final extension at 72 °C for 10 min. Gel electrophoresis confirmed specific amplification of OsKAT1 only in root samples under both conditions, supporting root-predominant expression consistent with Fig. 1A–E and *SI Appendix*, Fig. S1. The lower panel shows amplification of OsActin (26 cycles) to justify equal amount of template input. (B) RT-PCR analysis of OsKAT1 expression in various tissues from field-grown mature plants (Nipponbare), following previously described methods (8). Three biological replicates were analyzed for flag leaves, internodes, mature leaves, leaf sheath, caryopses, and rachides. Expression was also detected in internodes and rachides, suggesting that OsKAT1 may be expressed in specific aerial tissues during later developmental stages.

Legend to Supplementary movies 1 and 2:

Dynamic interactions between a classical C1-terminus and the C-linker were predicted with molecular dynamics simulation using Gromacs. 8-second movies were captured at time step of 100ns. Movie 1: front view; Movie 2: end view. Red: C1-terminus; Cyan: C-linker; Violet: S6 helix; Sky blue: S4 helix; Yellow: S4-S5 linker; S1, S2, S3 and S5 helices were shown in green.

Table S1. Primers used in this study

#	Primer description		Primer usages
**Primers used for construction of electrophysiological plasmid vectors**			
Primer 1	*OsKAT1-Xho* I-P1	CTCGAGATGCCACGTTCTTCTCGTATG	
Primer 2	*OsKAT1-Not* I-P2	GCGGCCGCTTATACGTTCACTTGCTGAAGGTTG	Construction for pCI-*OsKAT1* and pCI-*OsKAT1*+C1 (with primers 1- 2)
Primer 3	*AtKAT1-Xho* I-P1	CTCGAGATGTCGATCTCTTGGACTCG	
Primer 4	*AtKAT1-Not* I-P2	GCGGCCGCTCAATTTGATGAAAAATACAAATGATCA	Construction for pCI-*AtKAT1* with primers3-4
Primer 5	*AtKAT1*-M1	TCTAGATATCCGCAGTATTTGAG	
Primer 6	*AtKAT1*-M2	TCTAGAACATCGCTGATGAGTG	Construction for pCI-*AtKAT1△C1* with primers 3-6
Primer 7	*OsKAT1-Mlu* I-P1	tagcctcgagaattcACGCGTATGCCACGTTCTTCTCGTATGAA	
Primer 8	*OsKAT1+OsAKT1C*-P3	GCAACCTTCAGCAAGTGAACGTAGAGCAGAAGGAGAACAGTGTAATGGCTG	
Primer 9	*OsKAT1+OsAKT1C*-P4	CAGCCATTACACTGTTCTCCTTCTGCTCTACGTTCACTTGCTGAAGGTTGC	
Primer 10	*OsAKT1-Not* I-P2	tcatgtctgctcgaaGCGGCCGCCTAGCTCTTGCCTTTCATCTTCTCT	Construction for pCI-*OsKAT1+C2* with primers 7-10
Primer 11	*AtKAT1*+ANK-P3	attggcaaatcCTGTTGCCCTCTAAGTTTCATGAA	
Primer 12	*OsAKT1* ANK-P1	gggcaacagGATTTGCCAATTACACTCTGTTTTG	
Primer 13	*OsAKT1* ANK-P2	ttgctattgaACTCGCCGTCGCCGCCTT	
Primer 14	*AtKAT1*+ANK-P4	gacggcgagtTCAATAGCAATAGATGATTCGAATACTAG	
Primer 15	*AtKAT1-Not* I-P2	tcatgtctgctcgaaGCGGCCGCTCAATTTGATGAAAAATACAAATGATCA	Construction for pCI-*OsKAT1+C1+ANK* with primers 7, 11-15
Primer 16	*OsCBL1-Bgl* II-P1	cgctcaactttggcAGATCTATGGGGTGCTTCCAGTCGACGGCGAG	
Primer 17	*OsCBL1-Spe* I-P2	agatcctagtcagtcACTCGTTCATGTGACGAGATCATCAACCTC	Construction for pT7TS-*OsCBL1* with primers 16-17
Primer 18	*OsCIPK23-Bgl* II-P1	cgctcaactttggcAGATCTATGAGCGTGTCGGGCGGGAGGACG	
Primer 19	*OsCIPK23-Spe* I-P2	agatcctagtcagtcACTCGTTCACGGTGACCTCCGATGCTGGATC	Construction for pT7TS-*OsCIPK23* with primers 18-19
**Primers used for *OsKAT1* mutant plants**			
Primer 21	*OsKAT1* Spacer	CCTCCCAAGCTCAGGAAATACCT	
Primer 22	Mut-F	GTATGAATCTGTGGCCGCATTG	Construction and identification for *OsKAT1* mutant lines with
Primer 23	Mut-R	CGACATCGTCCACGACAAAAGG	primers 21-23
**Primers used for *OsKAT1* overexpression plants, RT-qPCR and in situ PCR**			
Primer 24	*OsKAT1-Xma* I-P1	GTCCCCGGGATGCCACGTTCTTCTCGTATGAATCTGTG	
Primer 25	*OsKAT1-Sac* I-P2	GTCGAGCTCTTATACGTTCACTTGCTGAAGGTTGCTTCTG	Construction for pUN1301-*OsKAT1* with primers 24-25
Primer 26	*OsKAT1*-qF	CCAGCATTTCTCACTACCTCTTC	
Primer 27	pUN1301-P2	GGCTTTACACTTTATGCTTCCGG	Identification for OE insert lines with primers 26-27
Primer 28	*OsKAT1*-qR	GTTGCACAGTACACCTATCTCC	Transcript abundance identification / *in situ* PCR with primers 26 and 28
Primer 29	*OsActin*-qF	TGGTCGTACCACAGGTATTGTGTT	
Primer 30	*OsActin*-qR	AAGGTCGAGACGAAGGATAGCAT	
**Primers used for *OsKAT1* Size identification**			
Primer 31	*OsKAT1*-P1	ATGCCACGTTCTTCTCGTATGAATCTGTGGC	
Primer 32	*OsKAT1*-P2	TTATACGTTCACTTGCTGAAGGTTGCTTCTGATGATGTC	
Primer 33	*OsKAT1*-3'	TTTTTTTTTTTTTTTTTTTTTTTTTTTTTTT	*OsKAT1* Size identification with primers 31-32, or primers 31and 33
**Primers used for eGFP fusion**			
Primer34	*OsKAT1* fusion eGFP-P4	ccttgctcaccatTACGTTCACTTGCTGAAGGTTGC	
Primer35	*OsKAT1* fusion eGFP-P3	gaacgtaATGGTGAGCAAGGGCGAGG	
Primer36	eGFP-NotI-P2	tcatgtctgctcgaaGCGGCCGCTCACTTGTACAGCTCGTCCATGC	Construction for pCI-*OsKAT1*+eGFP with primers 7, 34-36.
Primer37	*OsKAT1+C1* fusion eGFP-P4	gcccttgctcaccatATTTGATGAAAAATACAAATGATCACC	

Primer38	*OsKAT1+C1* fusion eGFP-P3	caaatATGGTGAGCAAGGGCGAGG	Construction for pCI-*OsKAT1+C1*+eGFP with primers 7, 36-38
Primer39	*OsKAT1+C2* fusioneGFP-P4	ctcaccatGCTCTTGCCTTTCATCTTCTCTG	
Primer40	*OsKAT1+C2* fusion eGFP-P3	aaaggcaagagcATGGTGAGCAAGGGCGAGG	Construction for pCI-*OsKAT1+C2*+eGFP with primers 7, 36, 39-40
**Primers used for *Arabidopsis* transformation lines**			
Primer41	*atskor*-LP	TCGAAATTTGAAAACACAGGC	
Primer42	*atskor*-LR	TGAAGAATCCGAATTCCAATG	Identification for *atskor* mutant
Primer43	*OsKAT1-Kpn*I-P1	atttggagaggacagggtaccATGCCACGTTCTTCTCGTATGAA	
Primer44	*OsKAT1-Bam*HI-P2	caggtcgactctagaggatccTTATACGTTCACTTGCTGAAGGTTG	Construction for pCambia1301-35S-NOS-*OsKAT1* with primers 43-44
Primer45	*OsKAT1+C1-Bam*HI-P2	caggtcgactctagaggatccTCAATTTGATGAAAAATACAAATGATCA	Construction for pCambia1301-35S-NOS-*OsKAT1*+C1 with primers 43 and
Primer46	*AtActin*-qF	AGCTCCTGGAATCCATGAAAC	
Primer47	*AtActin*-qR	CGGTGATCTCTTTGCTCATACG	qPCR for transcript abundance identification with primers 46-47
Primer48	*OsKAT1+C1*-qF	GCGATGCATGCTCATGCTGACGATG	
Primer49	*OsKAT1+C1*-qR	GAGCAGTGTCGGAAGTCGGATTCG	Ttranscript abundance identificationof*OsKAT1+C1* with primers 48-49
**Primers used for *OsKAT1* localization analyses in protein level**			
Primer50	*OsKAT1*pro-*Bam*HI-P1	gaattatctaagcttggatccAGAATTAAAGGTGTTGCAAGTGAGTT	
Primer51	*OsKAT1*pro-CDS-P2	tAATATTTCGGTCCAGGGCGA	
Primer52	*OsKAT1*pro-CDS-P3	cgccctggaccgaaatattATGCCACGTTCTTCTCGTATGAA	Construction for pCambia1301-*OsKAT1*promoter-OsKAT1CDS-eGFP with
Primer53	eGFP-NOS-pCAMBIA1301-P4	gggaaattcgagctcggtaccTCACTTGTACAGCTCGTCCATGC	Primers 34-35, 50-53.

Table S2. Shaker polypeptide accessions used in phylogenetic analyses

Name	Accession (NCBI)	Other name	Species	Isoforms	Gene symbol (NCBI)	Chromosome
AeK2.2 (m)	XP_020156780.1		Aegilops tauschii subsp. strangulata		LOC109742118	3D
AeK2.3	XP_040248167.1		Aegilops tauschii subsp. strangulata	XR_002227716.2 (mRNA)	LOC109741906	6D
BdK2.1	XP_010230608.1	BRADI_2g06750	Brachypodium distachyon (stiff brome)	XP_024315417.1 XP_024315418.1 XP_010230609.1	LOC100824787	2
BdK2.2 (m)	XP_003569842.2	BRADI_2g50260	Brachypodium distachyon (stiff brome)		LOC100845192	2
BdK2.3	XP_014757009.1	BRADI_3g09290	Brachypodium distachyon (stiff brome)	XP_024316194.1 XP_024316195.1 XP_014757012.1 XP_010234093.1	LOC100846215	3
HvK2.1	KAE8779374.1		Hordeum vulgare			
HvK2.2 (m)	KAE8818688.1		Hordeum vulgare			
HvK2.3	KAE8780447.1		Hordeum vulgare			
ObK2.2 (m)	XP_006644729.1		Oryza brachyantha (malo sina)		LOC102722537	1
ObK2.1	XP_015688137.1		Oryza brachyantha (malo sina)	XP_015688138.1 XP_015688140.1 XP_015688139.1 XP_015688141.1	LOC102711193	1
ObK2.3	XP_006647116.1		Oryza brachyantha (malo sina)		LOC102708684	2
OsK2.2 (m)	XP_015632943.1	OSNPB_010756700 Os01g0756700 LOC_Os01g55200	Oryza sativa Japonica Group (Japanese rice)		LOC4325143	1
OsK2.1	XP_015614092.1	OSNPB_010210700 Os01g0210700 LOC_Os01g11250	Oryza sativa Japonica Group (Japanese rice)	XP_015614100.1	LOC4325560	1
OsK2.3	XP_015623358.1	OSNPB_020245800 Os02g0245800 LOC_Os02g14840	Oryza sativa Japonica Group (Japanese rice)		LOC4328864	2
PhK2.3	XP_025809535.1	PAHAL_1G104500	Panicum hallii	XP_025809542.1 XP_025809551.1	LOC112887556	1
PhK2.2 (m)	XP_025817225.1	PAHAL_5G153800	Panicum hallii		LOC112893913	5
PhK2.1	XP_025816719.1	PAHAL_5G518300	Panicum hallii]		LOC112893541	5
PvK2.3k	XP_039834971.1	PVAP13_1KG176200	Panicum virgatum (switchgrass)	XP_039834975.1 XP_039834982.1	LOC120695798	1K
PvK2.3n	XP_039788930.1	PVAP13_1NG132900	Panicum virgatum (switchgrass)		LOC120655247	1N
PvK2.1k	XP_039845996.1		Panicum virgatum (switchgrass)		LOC120705619	5K
PvK2.1n	XP_039808605.1	PVAP13_5NG052032	Panicum virgatum (switchgrass)	XP_039808606.1 XP_039808607.1	LOC120672334	5N
PvK2.2n (m)	XP_039808087.1	PVAP13_5NG503500	Panicum virgatum (switchgrass)		LOC120671890	5N
SiK2.3	XP_004951327.1	SETIT_016455mg	Setaria italica (foxtail millet)		LOC101784878	I

SiK2.1	XP_022682667.1		Setaria italica (foxtail millet)	XR_002677822.1 XR_002677821.1 XR_002677819.1 XR_002677820.1 XR_002677818.1 (mRNAs)	LOC101769500	V
SiK2.2 (m)	XP_004970003.1	SETIT_000905mg	Setaria italica (foxtail millet)		LOC101758676	V
SvK2.1	XP_034594027.1	SEVIR_5G146600v2	Setaria viridis	XP_034594028.1 XP_034594029.1 XP_034594030.1	LOC117855762	5
SvK2.2 (m)	XP_034594133.1	SEVIR_5G326800v2	Setaria viridis		LOC117855845	5
SbK2.2 (m)	XP_002456376.1	SORBI_3003G300600	Sorghum bicolor (sorghum)	XP_021313277.1	LOC8072092	3
SbK2.3	XP_002453600.1	SORBI_3004G107500	Sorghum bicolor (sorghum)	XR_002452445.1 (mRNA)	LOC8085586	4
TdK2.1a	XP_037404828.1		Triticum dicoccoides	XP_037404829.1	LOC119267534	3A
TdK2.2a (m)	XP_037406682.1		Triticum dicoccoides		LOC119269037	3A
TdK2.1b	XP_037412182.1		Triticum dicoccoides	XP_037412183.1	LOC119275441	3B
TdK2.2b (m)	XP_037417926.1		Triticum dicoccoides		LOC119281530	3B
TdK2.3a	XP_037446619.1		Triticum dicoccoides		LOC119316403	6A
TdK2.3b	XP_037455409.1		Triticum dicoccoides	XP_037455410.1	LOC119325785	6B
ZmK2.1	NP_001105161.1	ZEAMMB73_Zm00001d039386	Zea mays		LOC542053	3
ZmK2.3-1	NP_001105240.3	ZEAMMB73_Zm00001d053446	Zea mays	XP_008677784.2 XR_004857631.1 (mRNA)	LOC542145	4
ZmK2.3-2	XP_008645574.1	ZEAMMB73_Zm00001d016160	Zea mays		LOC103626996	5
ZmK2.2 (m)	XP_008657217.1		Zea mays		LOC103636639	8
ZoK2-3a (m)	XP_042467449.1	ZIOFF_018945	Zingiber officinale		LOC122050623	3A
ZoK2-3b (m)	XP_042472397.1	ZIOFF_022406	Zingiber officinale		LOC122055065	3B
ZoK2-7b (m)	XP_042415042.1	ZIOFF_050277	Zingiber officinale		LOC122004183	7B
NtoK5.1 (d)	XP_009597240.1		Nicotiana tomentosiformis	XP_033511256.1 XP_009597241.1	LOC104093217	
NtaK5.1 (d)	XP_016443606.1		Nicotiana tabacum	XP_016443607.1 XP_016443608.1	LOC107768944	
AtGORK		At5g37500	Arabidopsis thaliana			5
AtSKOR		At3g02850	Arabidopsis thaliana			3
AtKC1		At4g32650	Arabidopsis thaliana			4
HuK4.1 (d)	XP_021276300.1		Herrania umbratica		LOC110410762	
QsK4.1 (d)	XP_023886332.1	CFP56_45492	Quercus suber		LOC111998467	
NsK4.1 (d)	XP_009767540.1		Nicotiana sylvestris	XP_009767541.1 XP_009767542.1 XP_009767543.1 XP_009767544.1	LOC104218690	
InK4.1 (d)	XP_019157682.1		Ipomoea nil		LOC109154314	
AtAKT2		At4g22200	Arabidopsis thaliana			4
PsK3.1 (d)	XP_026437959.1	C5167_027024	Papaver somniferum	XP_026437958.1	LOC113336187	
AtAKT5		At4g32500	Arabidopsis thaliana			4
AtSPIK		At2g25600	Arabidopsis thaliana			2
AtAKT1		At2g26650	Arabidopsis thaliana			2
AoK1.1 (m)	XP_020259216.1	A4U43_C03F29960	Asparagus officinalis	XR_002245639.1 (mRNA)	LOC109835651	
AtKAT1		At5g46240	Arabidopsis thaliana			5
AtKAT2		At4g18290	Arabidopsis thaliana			4

SI References

1. T. H. Nguyen, *et al*., A dual role for the OsK5.2 ion channel in stomatal movements and K^+ loading into xylem sap. *Plant Physiol*. **174**, 2409-2418 (2017).
2. J. Miao, *et al*., Targeted mutagenesis in rice using CRISPR-Cas system. *Cell Res.* **23**, 1233-1236 (2013).
3. X. X. Hu, *et al*., Expanding the range of CRISPR/Cas9 genome editing in Rice. *Mol. Plant* **9**, 943-945 (2016).
4. X. L. Ma, *et al*., A robust CRISPR/Cas9 system for convenient high-efficiency multiplex genome editing in monocot and dicot Plants. *Mol. Plant* **8**, 1274-1284 (2015).
5. Z. Wang, *et al*., A practical vector for efficient knockdown of gene expression in rice (*Oryza sativa* L.). *Plant Mol. Biol. Rep.* **22**, 409-417(2004).
6. X. H. Huang, Z. X. Wang, J. L. Huang, S. B. Peng, D. L. Xiong, Mesophyll conductance variability of rice aquaporin knockout lines at different growth stages and growing environments. *Plant J.* **107**, 1503-1512 (2021).
7. Y. N. Huang, *et al*., The rectification control and physiological relevance of potassium channel OsAKT2. *Plant Physiol*. **187**, 2296-2310 (2021).
8. T. Obata, *et al*., Rice shaker potassium channel OsKAT1 confers tolerance to salinity stress on yeast and rice cells. *Plant Physiol*. **144**, 1978-1985 (2007).
9. A. Nakamura, A. Fukuda, S. Sakai, Y. Tanaka, Molecular cloning, functional expression and subcellular localization of two putative vacuolar voltage-gated chloride channels in rice (*Oryza sativa* L.). *Plant Cell Physiol.***47**, 32-42 (2006).
10. F. Gaymard, *et al*., Identification and disruption of a plant shaker-like outward channel involved in K^+ release into the xylem sap. *Cell***94**, 647-655 (1998).
11. Q. X. Tian, *et al*., Rice shaker potassium channel OsAKT2 positively regulates salt tolerance and grain yield by mediating K^+ redistribution. *Plant Cell Environ*. **44**, 2951-2965 (2021).
12. M. Jabnoune, *et al*., Diversity in expression patterns and functional properties in the rice HKT transporter family. *Plant Physiol.* **150**, 1955-1971 (2009).
13. N. Yamaji, J. F. Ma, Spatial distribution and temporal variation of the rice silicon transporter Lsi1. *Plant Physiol.***143**, 1306-1313 (2007).
14. T. G. Andersen, *et al*., Grafting *Arabidopsis*. *Bio-Protocol* **4**, e1164 (2014).
15. M. Han, W. Wu, W.H. Wu, Y. Wang, Potassium transporter KUP7 is involved in K^+ acquisition and *translocation* in *Arabidopsis* root under K^+-limited conditions. *Mol. Plant***9**, 437-446 (2016).
16. S. Y. Yang *et al*., Internal ammonium excess induces ROS-mediated reactions and causes carbon scarcity in rice. *BMC Plant Biol.* **20**, 143 (2020).
17. D. L. Hao, *et al*., Functional characterization of the *Arabidopsis* ammonium transporter AtAMT1;3 with the emphasis on structural determinants of substrate binding and permeation properties. *Front. Plant Sci.* **11**, 571 (2020).
18. L. Wang, *et al*., The S1-S2 linker determines the distinct pH sensitivity between ZmK2.1 and KAT1. *Plant J.* **85**, 675-685 (2016).

19. G. Z. Yang, H. Sentenac, A.-A. Véry, Y. H. Su, Complex interactions among residues within pore region determine the K^+ dependence of a KAT1-type potassium channel AmKAT1. *Plant J.* **83**, 401- 412 (2015).
20. L. H. Wegner, K. Raschke, Ion channels in the xylem parenchyma of barley roots. *Plant Physiol.* **105**,799-813 (1994).
21. S. K. Roberts, M. Tester, Inward and outward K^+-selective currents in the plasma membrane of protoplasts from maize root cortex and stele. *Plant J.* **8**, 811-825 (1995).
22. T. X. Chen, et al., Overexpression of OsGS1;2 for improved nitrogen use efficiency and grain yield of rice: A field test. *Field Crop Res.* **303**, 109146 (2023).
23. T. Ehrhardt, S. Zimmermann, B. Müller-Röber, Association of plant K^+ in channels is mediated by conserved C termini and does not affect subunit assembly. *FEBS Lett.* **409**, 166-170 (1997).
24. G. Pilot, R. Pratelli, F. Gaymard, Y. Meyer, H. Sentenac, Five-group distribution of the Shaker-like K^+ channel family in higher plants. *J. Mol. Evol.***56**, 418-434 (2003).
25. R. C. Edgar, MUSCLE: multiple sequence alignment with high accuracy and high throughput. *Nucleic Acids Res.***32**, 1792-1797 (2004).
26. J. Castresana, Selection of conserved blocks from multiple alignments for their use in phylogenetic analysis. *Mol. Biol. Evol.***17**, 540-552 (2000).
27. M. Gouy, S. Guindon, O. Gascuel, SeaView version 4: A multiplatform graphical user interface for sequence alignment and phylogenetic tree building. *Mol. Biol. Evol.* **27**, 221-224 (2010).
28. M. D. Clark, G. F. Contreras, R. Shen, E. Perozo, Electromechanical coupling in the hyperpolarization-activated K^+ channel KAT1. *Nature* **583**,145-149 (2020).
29. S. Y. Li et al., Cryo-EM structure of the hyperpolarization-activated inwardly rectifying potassium channel KAT1 from Arabidopsis. *Cell Res.* **30**, 1049-1052 (2020).
